# Essential oil nanoemulsions enhance protection of stored tobacco against *Lasioderma serricorne*

**DOI:** 10.1038/s41598-026-45107-x

**Published:** 2026-04-09

**Authors:** Magdy A. Massoud, Abdelfattah S. A. Saad, Hassan A. Mesbah, Rasha A. Zinhoum, Marwa I. Mackled, Adel M. Khider, Khaled H. M. Abdel-Rheim

**Affiliations:** 1 Plant Protection Department, Faculty of Agriculture (Saba-Basha), Alexandria University, Alexandria, 21531 Egypt; 2 Stored Grains and Product Pests Research Department, Plant Protection Research Institute, Agricultural Research Center, Cairo, Egypt

**Keywords:** Stored tobacco, Fumigant toxicities, Contact activity, *Lasioderma serricorne* (F.), Physicochemical characterization, Nanoemulsion, Kinetic stability, Essential oil, Biological techniques, Biotechnology, Chemical biology, Environmental sciences, Plant sciences, Zoology

## Abstract

Essential oils present a promising botanical insecticide for sustainable pest control. Sourced from plants such as cinnamon, clove, and peppermint, these biocompounds are eco-friendly alternatives to conventional pesticides, employing unique modes of action. Their application is of relevance to the global tobacco industry, which suffers significant annual losses from insects during storage and handling. While several control strategies exist, heavy dependence on synthetic insecticides has resulted in pest resistance, environmental contamination, and harm to non-target organisms. This study explores how nanoemulsification can enhance the insecticidal efficacy of these essential oils. Using a high-energy ultrasonication method (20 kHz, 60% amplitude, 120 W, below 30 °C via ice-water bath, successfully, monodisperse nanoemulsions fabricated stable (NEs, surfactants Tween 80: Span 20 at 4:1 ratio). Transmission electron microscopy and dynamic light scattering revealed spherical particles with diameters between 15.77 and 46.27 nm (TEM) and 18.3–51.7 nm (DLS, PDI < 0.20). The formulations demonstrated kinetic, long-term, thermal, and centrifugal stability, possessed acidic pH values (4.50–5.60), and exhibited satisfactory physicochemical properties, including a zeta potential ranging from − 30.5 to − 50.5 mV. Bioassays for contact and fumigant toxicity showed that nanoemulsification significantly increased insecticidal potency against both adult and larval stages, with median lethal concentration (LC₅₀) values for the NEs 9.4 to 162.1 times lower than those of their pure essential oil counterparts. Clove NE was the most potent formulation overall, especially against larvae (LC₅₀ = 32.71 ppm). The NEs also produced steeper probit slopes than the pure oils, indicating a faster and more uniform toxic response. Mechanistic differences between life stages are discussed in the context of cuticle thickness and metabolic susceptibility. In simulated storage trials on treated tobacco leaves, NEs applied at a 25-fold lower concentration (40 ppm) achieved 70–90% mortality after 72 h, comparable to pure oils applied at 1000 ppm. Statistical analysis confirmed that concentration and exposure time were the primary factors influencing mortality, with a smaller but significant effect from the type of essential oil and no significant interaction effects. Fumigant toxicity bioassays further confirmed the superior efficacy of the NEs, with enhancement factors ranging from 17.1 to 143.5-fold over pure oils, as measured by reduced LC₅₀ values, and statistical significance was confirmed by non-overlapping 95% confidence intervals. Adults were consistently more susceptible than larvae across cinnamon and peppermint nanoemulsion treatments. The findings strongly support the use of clove, cinnamon, and peppermint nanoemulsions as highly effective, eco-friendly botanicals for protecting stored tobacco. Stability conclusions are limited to laboratory physicochemical indices, and future work should address real-world conditions and non-target effects.

**Supplementary Information:**

The online version contains supplementary material available at 10.1038/s41598-026-45107-x.

## Introduction

Tobacco (*Nicotiana tabacum*) is an agricultural commodity that is processed and traded in the same way as food products^1,2^. It is a member of the Solanaceae family and is one of the most important industrial crops in the world^3,4^. In addition to its leaf being used primarily for cigarettes and other tobacco products, tobacco is an oilseed crop that can be used to produce biofuels and sustainable biomass. The tobacco plant may have medicinal uses, especially when the nicotine component is isolated^5^. About 38% of the world’s tobacco is grown in China; about 25% is grown in the United States, Brazil, and India combined; and 15% is grown in Turkey, Indonesia, Zimbabwe, Italy, Greece, Argentina, and Malawi; thus, about 80% of the world’s tobacco is produced in 11 countries, with small amounts grown in about 70 other developing countries^6,7^. Recently, the international tobacco industry has stressed the economic relevance of tobacco^8^, where local governments rely heavily on cigarette manufacturing as a major source of cash. In 2020, Egypt ranked 17th globally in terms of raw tobacco imports, according to the Institute of National Planning^9^. Egypt imported 11.32 million kg of unmanufactured tobacco in 2023, valued at $60.09 million from, China and India; furthermore, the USA, Brazil, Italy, and Syria^10^.

Insect pests infest tobacco leaves during curing, handling, and storage, causing significant yearly losses for the worldwide tobacco industry, which estimates that there is a 3% loss of production due to the presence of these insects in storage^11^, from a global market size worth over 814 billion USD in 2018^12^. *Lasioderma serricorne*(F.) (Coleoptera: Anobiidae) is a tobacco or cigarette beetle that predominantly infests tobacco in its various forms, including processed tobacco and products such as cigarettes and cigars^13^. The economic repercussions of this pest are significant, resulting in annual losses of 0.7%−1.0% in worldwide tobacco production^14^. While the adult tobacco beetles do not feed, the larvae and mature cigarette beetles burrow into dry tobacco, causing spoilage due to significant damage^15^ and creating large holes in the tobacco, thereby contaminating the product with excessive frass production.

The tobacco companies employ various methods to avoid insect infestations in stored tobacco. Most often, these strategies involve the use of synthetic insecticides and fumigants. Phosphine usage for decades in controlling tobacco beetles (*L. serricorne*) has led to negative effects, including pesticide resistance^16^. It also disrupts natural enemy biological control, causing problems with the environment and health. Various nonchemical pest management methods have had mixed results^17^. Thus, it is crucial to identify environmentally friendly methods for controlling *L. serricorne* that do not compromise product quality.

Using botanical insecticides is one potential strategy. The biocompounds made from different plant species provide a more sustainable and eco-friendly method of managing pests^18^. Additionally, according to Vilas-Boas et al^19–22^., the biocompounds have a novel mode of action, are biodegradable, have low toxicity to mammals, and cause less environmental harm. Additionally, insect pests face difficulties in developing resistance to these biocompounds. In recent years, the use of essential oils in pest management has increased. Many researchers have examined how plant essential oils (EOs) attract or repel tobacco beetles and kill pests on touch and through fumigation^23–27^. Several researchers have reported the compositional profile of essential oils from diverse regions around the globe (see Supplementary Table S4). Peppermint (*Mentha piperita*) essential oil has two primary components: menthol and menthone^28–31^. Also, clove (*Syzygium aromaticum*) essential oil contains high levels of the phenolic monoterpenes eugenol and eugenol acetate^32–34^. Additionally, cinnamon (*Cinnamomum zeylanicum*) essential oil contains cinnamaldehyde as the main component found^35–37^.

The essential oil of peppermint has demonstrated significant insecticidal and repellent efficacy against several stored insect pests, including *Sitophilus oryzae*^38–40^, *Tribolium castaneum*, and *L. serricorne*^41^. Clove essential oil has demonstrated potent insecticidal and repellent properties against several stored insect pests, including *Sitophilus zeamais*^42^, *Rhyzopertha dominica*^43^, *S. oryzae*^44^, and *T. castaneum*^45^. Cinnamon essential oil was effective against *Sitophilus granarius*^46^, and *Callosobruchus chinensis*^47^, *L. serricorne*^48^.

However, the share of biopesticide-based essential oils in the global pesticide market remains extremely small because of their instability, photosensitivity, and quick oxidation, which reduce their effectiveness in pest control^49^. As a result, they must be handled with caution and under suitable conditions^50^. To solve this problem, essential oils can be turned into nanoemulsions, which help make the active ingredients less likely to evaporate, mix better with water, and react with other substances. Nanoemulsion (NE) is an Oil-in-water (O/W) or water-in-oil (W/O) systems with tiny, spherical droplets less than 200 nm in size^51–57^. It reduces the hydrophobicity, volatility, and reactivity of bioactive compounds in essential oils. Thereby, researchers have investigated using nanoemulsion formulations as a different way to make essential oils more available and effective^39,58^. The stability of these nanoscale systems is paramount for their efficacy. Recent advances in colloid science have highlighted the critical role of electrostatic repulsions, even in systems employing nonionic surfactants, where charged species or interactions at the oil-water interface can generate a significant surface potential, preventing droplet coalescence^59–61^. Preliminary studies have shown that loading EOs into nanoemulsions using the sonication technique increases the toxicity of EOs against stored product insect pests. Jasman et al.^62^. used contact toxicity and fumigant toxicity bioassays to test the insecticidal activity of essential oils from lemongrass and wild mint, as well as their respective nanoemulsions, against the red flour beetle. They found that the nanoemulsion formulations increased biological efficacy by 40–45%, most likely due to improved penetration through the insect cuticle and increased surface area. In another study, the toxicity of clove essential oil nanoemulsion against the red flour beetle population was investigated by^63^, and they reported that the high toxicity of clove nanoemulsion is due to the alterations in metabolism. Additionally, EO-based NEs from *Mentha longifolia*, *Carlina acaulis*, and *Hazomalania voyronii* were investigated by^61,64,65^ for their potential as insecticides against *S. oryzae* on barley, oats, and maize kernels and they found that the application of EO-based NEs from *H. voyronii* and *C. acaulis* can effectively control the significant impact of EO-based NE against *S. oryzae* adults on barley. In the same context, the effect of *Vitex negundo* oil and its nanoemulsion formulation was examined by^66^, who found that the nanoemulsion formulation’s lethal dose 50 values for fumigant toxicity and contact toxicity were lower than those of crude oil against *S. oryzae* and *T. castaneum*. These results also agree with^67^, who investigated the fumigant toxicity of the prepared peppermint nanoemulsion and piperitone combinations and reported that the nanoemulsion was extremely efficient against *S. oryzae*, *T. castaneum*, and *Callosobruchus maculatus* adults.

As far as we know, no literature exists on the insecticidal properties of an improved formulation of cinnamon, clove, and peppermint essential oils against tobacco beetle adults and larvae. So, the purpose of this study was to formulate stable nanoemulsions utilizing surfactants, further study the physicochemical properties of the formulation, specify the effect of the prepared nanoemulsion on increasing the effectiveness, and aid in the transition from preventative, high-dose applications to low-dose. Thus, this study’s findings are expected to provide technical support for the development of plant-based insect control strategies and utilization of efficient formulations for stored tobacco protection and offer promising alternatives to conventional synthetic pesticides.

## Materials and methods

Essential oils of cinnamon (*Cinnamomum zeylanicum*), clove (*Syzygium aromaticum*), and peppermint (*Mentha piperita*) were sourced from the Medicinal Plants and Extracts Unit at the National Research Centre (Egypt). The cinnamon oil was derived from the inner bark, clove oil from dried flower buds, and peppermint oil from the aerial parts (leaves and stems). In each case, the oils were produced by steam distillation.

### Insect rearing

One hundred adult insects from the Stored Grain Insect Pests Department of the Plant Protection Research Institute, ARC, Dokki, Giza, Egypt, were used to begin the cultures of the cigarette beetle, *L. serricorne*. At the Department of Stored Grains and Product Pests Research, Plant Protection Research Institute, Agricultural Research Center, Alexandria, Egypt, the insects were raised on corn: beer yeast: tobacco leaves with ratio 90: 5: 5, respectively as food in flat plastic plates measuring 35 × 18 × 25 cm. In an electrical incubator, the rearing process was conducted under control conditions at 28 ± 2 °C; 65 ± 5% relative humidity and photoperiod of 14 L: 10D. Cocoons show up as small chambers on the petri dish sidewalls, which are easy to observe. When four weeks had passed, the new offspring began to show. Every day, dishes were checked for adult emergence. Males and females were kept apart throughout the pupal stage as described by^68^.

### Preparation of nanoemulsion

Nanoemulsions (NEs) were formulated using a high-energy ultrasonication method^58^. Tween 80 and Span 20 were selected as non-ionic surfactants due to their established safety, emulsification efficiency, and compatibility with essential oils^32,58^.

Rationale for ratio: The 4:1 Tween 80:Span 20 ratio was chosen based on preliminary screening, where ratios of 3:1, 4:1, and 5:1 were tested for emulsion clarity, stability, and minimal phase separation over 7 days (See Supplementary Table S1). The 4:1 blend yielded the best results, consistent with published nanoemulsion protocols^58^. Nanoemulsions contained 4% essential oil, 20% (w/w) surfactant blend (Tween 80 + Span 20, 4:1), and deionized water (76%). The mixture was pre-emulsified via magnetic stirring at 500 rpm for 45 min. Ultrasonication: A bandelin Sonopuls ultrasonicator-Germany (20 kHz, 120 W, 60% amplitude) was used for 30 min (1-min pulses with 30 s rest) while temperature was maintained < 30 °C using an ice-water bath.

### Characterization of prepared nanoemulsion

#### Droplet size analysis and surface morphology

The produced nanoemulsion of cinnamon, clove, and peppermint were examined for the average particle size as well as the morphological analysis. The morphology of a nano-formulation was seen, and droplet size was examined using a TEM (transmission electron microscope) (JEOL JEM-1400Plus, Japan)^69^. Once an appropriate dilution was made, samples were put on carbon-coated TEM grids, and a drop of 2% phosphotungstic acid was added. A filter paper was used to blot the extra liquid for two minutes to ensure that the stain layer is thin enough for the electron beam to penetrate and that it doesn’t form a thick, crystalline layer that would obscure the sample’s details. Before being observed, the sample was let dry at room temperature for ten minutes to allow the thin film of stain to solidify and form a stable, amorphous “cast” of the samples structure.TEM micrographs were obtained using an electron microscope equipped with a tungsten source and running at 80 kV. TEM micrographs were aquired at 50 K magnification. Mean hydrodynamic diameter and polydispersity index (PDI) were measured via DLS (Malvern Zetasizer Nano ZS) at 25 °C after diluting samples 1:100 in deionized water. Cinnamon NE: 18.3 ± 2.1 nm, PDI 0.18; Clove NE: 48.2 ± 3.8 nm, PDI 0.19; Peppermint NE: 51.7 ± 4.2 nm, PDI 0.17. DLS data are summarized in Supplementary Table S2. (See Supplementary Table S2).

#### The physico-chemical properties of nano formulation

##### Zeta potential analysis

Zeta potential was measured using the Malvern Zetasizer Nano ZS (4700 model). Samples were diluted 1:100 deionized water, equilibrated for 5 min at 25 °C before measurement. using the procedure outlined by^70^. The zeta potential was measured by dispersing the samples in a cuvette filled with 20 µL of deionized water.

##### pH measurement

The pH value of NEs was measured with a pH meter (Adwa - AD8000), as mentioned by^45^, by placing the electrode in the sample and waiting for 5 min at room temperature without stirring to let the pH value settle.

#### Stability studies

Stability of NEs was tested over 90 days at 4 °C and 25 °C, with droplet size and phase separation monitored monthly (see Supplementary Table S3). Thermal stability was assessed via three freeze-thaw cycles (− 20 °C/25°C), and centrifugal stability by centrifugation at 10,000 rpm for 30 min. No aggregation or phase separation was observed under any condition.

### Toxicity bioassays

#### Contact toxicity bioassay using thin film residue

The contact toxicity of clove, cinnamon, and peppermint free EO and its formulated nanoemulsion to *L. serricorne* adults and larvae was determined using a direct contact assay as described by^71^. Volumes of 10, 15, 20, and 25 µL of the tested nanoemulsion were added using a micropipette into a 9 cm glass Petri dish containing 2 mL of acetone. Volumes applied were converted to ppm by calculating the mass of essential oil in each applied volume (accounting for NE/oil concentration) and dividing by the surface area (for Petri dishes) or by the weight of treated tobacco leaves. For example, 10 µL of 4% NE contains 0.4 mg EO; spread over a 9-cm dish (see Supplementary Method for full calculation details). These volumes were equivalent to 200, 300, 400, and 500 ppm for nanoemulsion, and 4500, 6750, 9000, and 11,250 ppm for the pure oil. The liquid was then distributed evenly over the dish surface. The solvent was allowed to evaporate (10 min). Twenty *L. serricorne* larvae (10–12 days old) and adults (3–4 days old) were then placed in separate Petri dishes. The control treatment consisted of dishes containing only the solvent. Five replicates were set up for each treatment. The number of dead larvae and adults was counted, and mortality percentages were calculated 72 hours after treatment, and LC_50_ values were calculated according to^72^.

#### Toxicity of treated tobacco leaves against adults and larvae of *L. serricorne*

Clove, cinnamon, and peppermint free EO and its formulated nanoemulsion formulations were combined with unmanufactured tobacco leaves (Virginia). The effects of the investigated free EO and its formulated nanoemulsion formulations on the mortality of *L. serricorne* adults and larvae using the leaf-dipping method, following the method described in^73^ with some modifications. Briefly, varying concentrations of nanoemulsion formulations (16, 24, 32, and 40 ppm) and (400, 600, 800, and 1000 ppm) for the free EO were mixed with fifty grams of fluecured tobacco leaves (Virginia) were frozen for 48 h at − 26 °C to eliminate any potential insect contamination. The leaves were dipped in each volume for 30 s and then evaporated (5 min) air-dried. Subsequently, the fluecured tobacco leaves were placed in 250 ml glass jars. Twenty larvae (10–12 days old) and adults (3–4 days old) from *L. serricorne* were transferred to the jars. Acetone served as a negative control. The experiment was conducted under the same conditions as mentioned above in the insect rearing section, each treatment was replicated five times. Mortality was documented daily up to 72 h.

#### Fumigation toxicity against adults and larvae of *L. serricorne*

The fumigant efficacy of Clove, cinnamon, and peppermint free EO and its formulated nanoemulsion formulations against *L. serricorne* was determined using a modified fumigant toxicity assay^74^. Briefly, glass jars (250 ml) were used as fumigation chambers. The nanoemulsion and its pure oil were tested at different concentrations for adults and larvae. For adults, nanoemulsion volumes of 0.5, 1, 3, and 5 µL (equivalent to 0.08, 0.16, 0.48, and 0.80 ppm) and their pure oil equivalents (2.1, 4.2, 12.6, and 21.0 ppm) were tested. For larvae, nanoemulsion volumes of 10, 15, 20, and 25 µL (equivalent to 1.60, 2.40, 3.20, and 4.00 ppm) and their pure oil equivalents (42, 63, 84, and 105 ppm) were used. Test compounds were administered by applying each dose to a filter paper (Whatman No. 1, 2 × 3 cm) attached to the underside of the jar lid. Compounds were administered in various concentrations of adults and larvae. To keep the insects from coming into direct touch with the investigated compounds, Vaseline was applied to the inside of the neck of each jar. The tops were snugly fastened onto the jars, each holding twenty individuals of *L. serricorne* (larvae 10–12 days old and adults 3–4 days old). In the absence of the studied nanoemulsions, control insects were maintained in the same environment. Five replicates were set up for each treatment. Dead larvae and adults were recorded after 72 h of exposure, and the percentage of mortality was calculated. The LC_50_ (lethal concentration) and LC_95_ values were calculated.

Vapor-phase concentrations were estimated based on the applied dose and chamber volume, assuming ideal distribution. Losses due to adsorption to glass and filter paper were not directly measured. For rigorous quantification, future studies should measure actual headspace concentrations using gas chromatography.

### Statistical analysis

 Dose-mortality data were subjected to Probit analysis using SPSS Statistics (Version 28, IBM Corp.)^75^, The lethal concentrations causing 50% and 95% mortality (LC₅₀ and LC₉₅) with their respective 95% confidence intervals (CIs) and slope (± standard error) were calculated. The goodness-of-fit of the probit model was assessed by the Pearson Chi-square test (*p* > 0.05 indicates a good fit). Statistical comparisons of toxicity between different formulations and life stages were conducted using the method of overlapping 95% confidence intervals. In addition to 95% CI overlap, pairwise statistical comparisons of LC₅₀ values were conducted using likelihood ratio tests and Student’s t-test on regression slopes (see Supplementery Table S5). If the 95% CIs of two LC₅₀ values did not overlap, they were considered significantly different at *p* < 0.05 level. Enhancement factors (EF) were calculated for three independent NE batches to assess inter-batch variability (see Supplementery Table S6). The Enhancement Factor of the nanoemulsion over the pure oil was calculated for each plant and life stage as follows:

**EF** = LC₅₀ (Pure Oil)/LC₅₀ (Nanoemulsion). An EF > 1 indicates the nanoemulsion is more potent.

Separate two-way ANOVAs were conducted using SPSS Statistics for each formulation type (nanoemulsion, pure essential oil) and exposure time (24, 48, 72 h) to examine the effects of essential oil type (cinnamon, clove, peppermint) and concentration (nanoemulsions: 16, 24, 32, 40 ppm; pure oils: 400, 600, 800, 1000 ppm) on mortality on treated tobacco leaves. The model included main effects and two-way interactions. When significant main effects were detected (*p* < 0.05), post-hoc pairwise comparisons were conducted using Tukey’s Honestly Significant Difference (HSD) test. Effect sizes were calculated as partial eta squared (η^2^_p_) and interpreted as small (0.01), medium (0.06), or large (0.14) following Cohen’s conventions.

## Results

### Physicochemical property characterization of the nano formulations: surface morphology and droplet size distribution

**Fig. 1:**
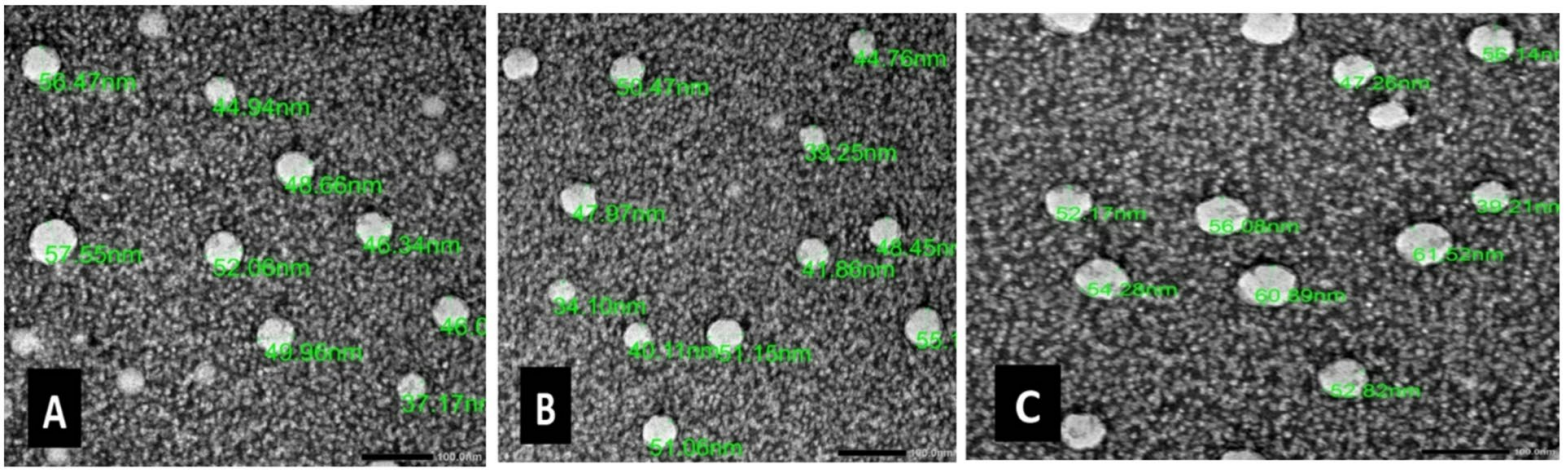
Transmission electron micrograph. (**A**) the cinnamon nanoemulsion, (**B**) clove nanoemulsion, and (**C**) peppermint nanoemulsion, showing the spherical shape of the prepared formulations *via* transmission electron microscopy (TEM) (MAG_X50k).

**Fig. 2:**
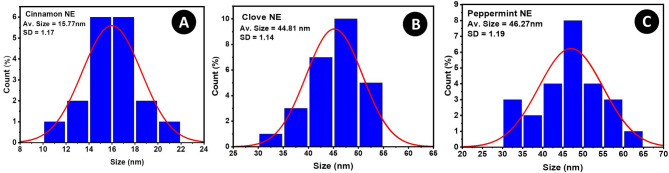
Particle size distribution for (**A**) the cinnamon nanoemulsion, (**B**) clove nanoemulsion, and (**C**) peppermint nanoemulsion, fitted with a log normal distribution function.

 Transmission electron microscopy (TEM) was used to validate the morphological and size distribution of the peppermint, clove, and cinnamon nanoemulsions, as seen in Fig. 1 (A, B, and C, respectively). DLS data confirmed the mean droplet sizes and monodispersity observed via TEM (Supplementery Table S2), addressing limitations of single-method particle sizing. The particle sizes are consistent, and their shapes are spherical. The estimated average particle size as seen in Fig. 2 (A, B, and C, respectively), cinnamon nanoemulsion is 15.77 nm with a standard deviation of 1.17 nm, and the coefficient of variation (CV) was 7.4%. Clove nanoemulsion measures 44.81 nm with a standard deviation of 1.14 nm, and CV was 2.54%; peppermint nanoemulsion is 46.27 nm with a standard deviation of 1.19 nm, and CV was 2.57%. All the particles are dispersed uniformly. To determine the average particle size for each formulation, the particle size distribution histogram was fitted to the log-normal distribution function^76^.

### Stability indices of cinnamon, clove, and peppermint nano emulsions

**Fig. 3:**
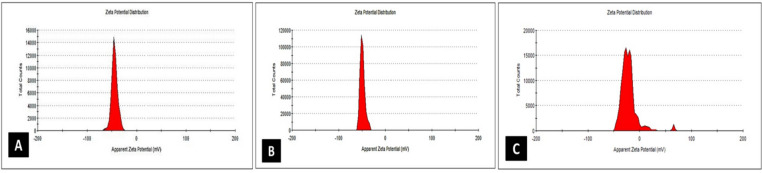
Zeta potential. (**A**) cinnamon nanoemulsion, (**B**) clove nanoemulsion, and (**C**) peppermint nanoemulsion all exhibited zeta potential values of −42.9, −50.5, and − 30.5 mV, respectively, indicating a strongly negative surface charge.

 Clove NE, cinnamon NE, and peppermint NE exhibited somewhat acidic pH levels (5.60, 4.50, and 5.52, respectively). There are notable differences in the zeta potential values of the three samples (Fig. 3), which indicate emulsion stability. Clove NE’s greatest absolute value of −50.5 mV indicated greater stability. No significant changes in droplet size or visible phase separation were observed after 90 days at 4–25 °C (Supplementery Table S3). Freeze-thaw and centrifugal stability tests also showed no aggregation, supporting the kinetic and physical stability of the NEs.

### Contact toxicity bioassay using thin film residue

The results described the contact toxicity assay (thin film residue method), highlighting the direct toxic effects of the tested essential oils and their nanoemulsion formulations against adults and larvae of *Lasioderma serricorne*.”. Probit analysis was successfully performed on mortality data to estimate lethal concentrations (LC₅₀ and LC₉₅) for all treatments against both adult and larval stages of *L. serricorne*. The corresponding 95% confidence intervals (CIs), slope values, and goodness-of-fit statistics are presented in Table 1. All probit models demonstrated an adequate fit to the data, as evidenced by non-significant chi-square values (*p* > 0.05 for all treatments). The toxicity profiles of the essential oil nanoemulsions (NEs) and their corresponding pure oils were directly compared by assessing the overlap of their 95% CIs. A lack of overlap between CIs indicates a statistically significant difference in potency at the LC₅₀ level. Nanoemulsions vs. Pure Oils, where, for every essential oil tested (cinnamon, clove, peppermint), the LC₅₀ 95% CI of the NE formulation did not overlap with the LC₅₀ 95% CI of its pure oil counterpart for either life stage. This confirms that all nanoemulsions were significantly more toxic than the unformulated oils. The magnitude of this enhancement is quantified by the enhancement factor (EF), which ranged from 9.4-fold (peppermint NE for adults) to 162.1-fold (clove NE for larvae). As for comparison among nanoemulsions: The LC₅₀ values among the three NEs varied. Against adults, the 95% CIs for cinnamon NE (299.45 ppm) and peppermint NE (289.16 ppm) showed considerable overlap, indicating no significant difference in their adulticidal potency. In contrast, the LC₅₀ CI for clove NE against adults (220.42 ppm) showed minimal or no overlap with the other two NEs, suggesting it may be the most potent adulticide. For larvae, clove NE was unequivocally the most potent larvicide, with an LC₅₀ of 32.71 ppm and a CI that did not overlap with the significantly higher LC₅₀ CIs of cinnamon NE (184.59 ppm) or peppermint NE (275.62 ppm).

The slopes of the probit regression lines differed markedly between formulations. The nanoemulsions produced consistently steeper slopes (range: 0.003 to 0.005 ± SE) compared to the very shallow slopes of the pure oils (range: 0.00004 to 0.0003 ± SE). This indicates a more homogeneous and rapid toxic response across the test population with increasing concentration of the NE, whereas the pure oils elicited a much more heterogeneous and gradual.

**Fig. 4:**
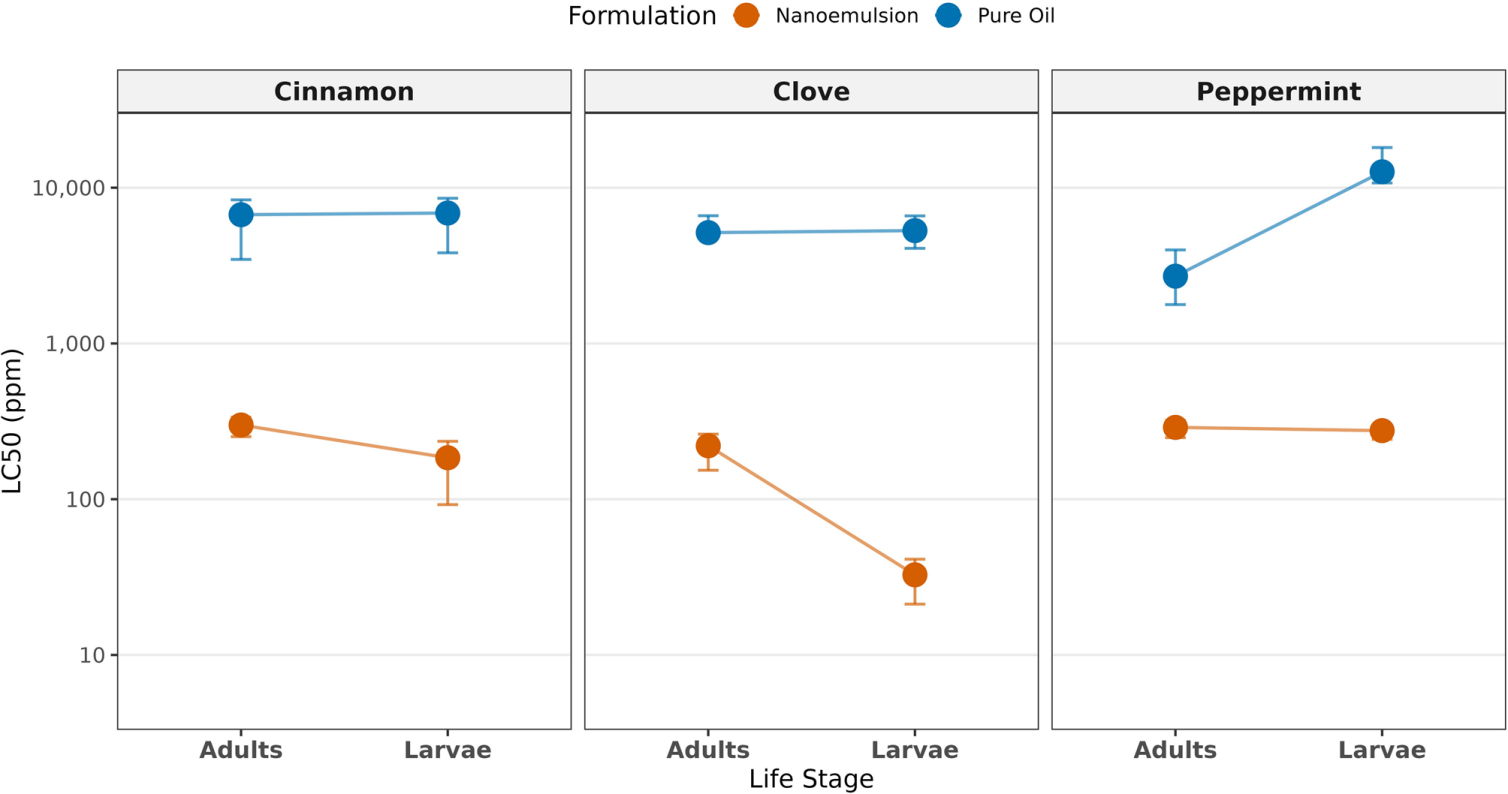
Paired dot plot with connecting lines that visually emphasize the differences in susceptibility between adult and larval stages for each tested formulation. The connecting lines immediately highlight which life stage was more affected per treatment. In Clove Nanoemulsion (NE), the difference was the most pronounced, with larvae being far more susceptible than adults. Cinnamon NE showed a moderate disparity between the stages. In contrast, Peppermint Oil exhibited an inverse relationship, with adults demonstrating greater susceptibility than larvae. Overall, the paired comparisons reveal consistent trends when contrasting the nanoemulsion formulations with the conventional oil treatment.

The relative susceptibility of larvae and adults to each treatment was determined by comparing the 95% confidence intervals (CIs) of their LC₅₀ values (Table 1; Fig. 4). Among the nanoemulsions (NEs), susceptibility differed significantly by life stage for two formulations: larvae were markedly more susceptible than adults to both Cinnamon NE (LC₅₀ CIs: 184.59 vs. 299.45 ppm) and Clove NE (LC₅₀ CIs: 32.71 vs. 220.42 ppm), as indicated by non-overlapping CIs. For Peppermint NE, however, the extensively overlapping CIs (larvae: 275.62 ppm; adults: 289.16 ppm) suggested comparable susceptibility. Conversely, for all three pure, unformulated oils, the LC₅₀ 95% CIs for adults and larvae showed complete or extensive overlap, demonstrating no significant difference in susceptibility between life stages. The slopes of the probit regression lines differed markedly between formulations. The nanoemulsions produced consistently steeper slopes (range: 0.003 to 0.005 ± SE) compared to the very shallow slopes of the pure oils (range: 0.00004 to 0.0003 ± SE). This indicates a more homogeneous and rapid toxic response across the test population with increasing concentration of the NE, whereas the pure oils elicited a much more heterogeneous and gradual. The efficacy of nanoemulsification was quantified by calculating the Enhancement Factor (EF), defined as the ratio of the LC_50_ of the pure oil to the LC_50_ of its nanoemulsion for the same life stage (Table 1). Nanoemulsification significantly potentiated the toxicity of all essential oils, with EFs ranging from 9.4 to 162.1. The greatest enhancement was observed for clove oil against larvae (EF = 162.1), followed by peppermint oil against larvae (EF = 45.9) and cinnamon oil against larvae (EF = 37.3). For adult stages, cinnamon and clove nanoemulsions showed similar, high enhancement (EF ~ 23), whereas peppermint nanoemulsion showed a more moderate but still substantial enhancement (EF = 9.4).

**Table 1:** Toxicological parameters of cinnamon, clove, peppermint free EO and its formulated nanoemulsions on *L. serricorne* adults and larvae exposed to thin film residues on glass 72 h. post-treatment.

Treatment	Stage	LC50 (ppm) [95% CI]	LC95 (ppm) [95% CI]	Slope ± SE (×10^−3^)	χ^2^ (*p*-value)	EF
Cinnamon NE	Adults	299.45 [252.12–335.81]	769.28 [659.03–983.92]	4 ± 1	0.827 (0.380)	22.4
	Larvae	184.59 [92.34–235.36]	652.19 [562.33–833.00]	4 ± 1	2.896 (0.235)	37.3
Cinnamon oil	Adults	6709.63 [3464.10–8354.61]	28115.47 [20078.33–64807.34]	0. 3 ± 0. 1	0.078 (0.962)	–
	Larvae	6878.32 [3819.38–8553.47]	28315.63 [20199.63–65470.18]	0. 3 ± 0.1	1.147 (0.563)	
Clove NE	Adults	220.42 [153.51–261.80]	650.60 [568.16–804.79]	4 ± 1	0.318 (0.853)	23.4
	Larvae	32.71 [21.17–41.18]	571.50 [504.51–683.06]	3 ± 1	0.232 (0.891)	162.1
Clove Oil	Adults	5147.75 [4897.55–6609.12]	46969.10 [45979.55–47960.01]	0. 04 ± 0. 03	0.063 (0.969)	–
	Larvae	5303.11 [4080.77–6592.68]	35607.24 [29290.55–37009.01]	0. 05 ± 0. 02	0.075 (0.963)	
Peppermint NE	Adults	289.16 [248.49–320.69]	684.70 [603.41–827.23]	4 ± 1	0.221 (0.895)	9.4
	Larvae	275.62 [242.40–302.00]	588.32 [473.83–699.93]	5 ± 1	0.627 (0.731)	45.9
Peppermint oil	Adults	2704.34 [1776.27–3987.01]	22547.19 [21074.02–23733.96]	0. 1 ± 0.04	0.646 (0.724)	–
	Larvae	12648.47 [10727.21–18107.03]	29447.06 [21984.18–53458.65]	0. 08 ± 0. 02	0.302 (0.860)	

CI = Confidence Interval. Slope values (×10^−3^) represent the PROBIT parameter estimate (B) from SPSS output multiplied by 1000. The corresponding standard error (SE) for each slope has also been multiplied by 1,000. χ^2^ represents the Pearson goodness-of-fit statistic with df = 2. All models showed adequate fit (*p* > 0.05). All models showed a good fit to the Probit model (*p* > 0.05 for Chi-Square GOF test). EF: Enhancement Factor = (LC₅₀ of Oil/LC₅₀ of NE). Significant differences in LC₅₀ values were inferred based on non-overlap of 95% confidence intervals (CIs).

### Toxicity of treated tobacco leaves against adults and larvae of *L. serricorne*

The mortality of *L. serricorne* adults exposed to cinnamon, clove, and peppermint essential oils (EOs) and their respective nanoemulsions (NEs) on treated tobacco leaves showed clear concentration- and time-dependent trends (Table 2). The formulated nanoemulsions demonstrated slightly enhanced insecticidal efficacy compared to their bulk oil counterparts, despite being applied at substantially lower concentrations. For all three plant extracts, mortality increased progressively with both increasing concentration and longer exposure periods from 24 to 72 h. Cinnamon NE at 40 ppm achieved 85.0% mortality after 72 h, matching the efficacy of the bulk cinnamon oil at 1000 ppm (85.0%). Notably, the NE at 32 ppm (78.0% mortality at 72 h) outperformed the bulk oil at 800 ppm (76.0%). Clove NE exhibited potent activity, with the 40-ppm formulation causing 86.0% mortality at 72 h. This was slightly higher than the 85.0% mortality caused by bulk clove oil at 1000 ppm after the same period. At 32 ppm, the clove NE (78.0% mortality) exhibited the same efficacy as the bulk oil at 800 ppm (78.0%), indicating a pronounced enhancement in bioavailability or activity. Peppermint NE also showed enhanced efficacy. The NE at 40 ppm resulted in 80.0% mortality at 72 h, which was comparable to the effect of bulk peppermint oil at 1000 ppm (79.0%). Furthermore, the NE at 32 ppm (74.0% mortality at 72 h) surpassed the bulk oil at 600 ppm (67.0%). At 24 h post-exposure, the nanoemulsions generally induced mortality comparable to or slightly higher than their corresponding bulk oils at much higher concentrations, highlighting their rapid action. This efficacy gap narrowed but remained evident at 48 and 72 h, with nanoemulsions consistently achieving high mortality at concentrations 25-fold lower (40 ppm NE vs. 1000 ppm bulk oil). No mortality was observed in the untreated control group throughout the 72-hour experiment.

**Table 2:** Mortality percentage of cinnamon, clove, and peppermint essential oils and their formulated nanoemulsions against adults of *L. serricorne* on tobacco leaves after 24, 48, and 72 h of exposure period.

Treatment	Conce (ppm)	Mortality (%) ± SE		
		24 h	48 h	72 h
Cinnamon (NE)	16	38.00 ± 0.99	40.00 ± 1.74	54.00 ± 1.77
	24	42.00 ± 3.92	55.00 ± 1.67	62.00 ± 1.96
	32	45.00 ± 4.74	60.00 ± 1.46	78.00 ± 1.50
	40	52.00 ± 6.58	72.00 ± 1.90	85.00 ± 0.89
Cinnamon oil	400	39.00 ± 0.97	44.00 ± 1.16	57.00 ± 0.93
	600	45.00 ± 1.227	59.00 ± 1.26	64.00 ± 1.93
	800	49.00 ± 1.077	62.00 ± 2.14	76.00 ± 1.66
	1000	55.00 ± 0.71	77.00 ± 1.40	85.00 ± 0.89
Clove (NE)	16	32.00 ± 1.21	44.00 ± 1.24	62.00 ± 2.66
	24	46.00 ± 1.49	54.00 ± 1.39	66.00 ± 2.87
	32	54.00 ± 0.75	62.00 ± 2.66	78.00 ± 1.50
	40	58.00 ± 1.81	76.00 ± 1.53	86.00 ± 1.39
Clove oil	400	39.00 ± 0.86	45.00 ± 1.07	53.00 ± 2.20
	600	49.00 ± 0.97	52.00 ± 1.33	56.00 ± 2.45
	800	52.00 ± 0.70	55.00 ± 2.27	78.00 ± 1.50
	1000	62.00 ± 1.11	71.00 ± 1.53	89.00 ± 1.02
Peppermint (NE)	16	32.00 ± 1.21	42.00 ± 0.78	58.00 ± 1.81
	24	44.00 ± 1.24	52.00 ± 1.32	68.00 ± 1.03
	32	52.00 ± 1.31	64.00 ± 1.39	74.00 ± 1.46
	40	62.00 ± 2.65	68.00 ± 1.03	80.00 ± 1.30
Peppermint oil	400	35.00 ± 0.86	40.00 ± 0.90	49.00 ± 1.85
	600	42.00 ± 0.97	56.00 ± 1.52	67.00 ± 1.18
	800	54.00 ± 1.37	58.00 ± 0.58	76.00 ± 1.41
	1000	63.00 ± 1.18	69.00 ± 1.21	79.00 ± 0.42
Cont	0	00.00 ± 00.00	00.00 ± 00.00	00.00 ± 00.00

NE = Nanoemulsion. Values are presented as mean ± standard error (SE).

The nanoemulsions (NEs) showed a dramatic increase in insecticidal potency against *L. serricorne* larvae compared to their non-emulsified essential oil counterparts (Table 3). Notably, a concentration of 40 ppm of cinnamon NE achieved 70% mortality after 72 h, exceeding the 63% mortality caused by 1000 ppm of pure cinnamon oil. Similarly, clove NE at 40 ppm (88% mortality) matched the performance of 1000 ppm clove oil (89%), despite using only 1/25th of the concentration. Peppermint NE at 40 ppm (90%) was equally effective as 1000 ppm peppermint oil (92%). For all agents, larval mortality showed a clear positive correlation with both concentration and exposure time. For instance, mortality from cinnamon NE rose from 24% at 16 ppm to 48% at 40 ppm after 24 h and further increased to 70% at the same concentration after 72 h, a trend consistent across all treatments. Among the nanoemulsions, peppermint NE was the most potent, producing the highest mortality in its concentration series at 72 h. Pure peppermint oil also showed the greatest efficacy among the unformulated oils, reaching 92% mortality at 1000 ppm. No mortality occurred in the untreated control group.

While direct statistical comparison between nanoemulsions and pure essential oils was precluded by their different concentration ranges, descriptive analysis suggests important formulation differences. Nanoemulsions achieved substantial mortality effects at concentrations (16–40 ppm) that are 10–25 times lower than those required for pure essential oils (400–1000 ppm) to produce comparable effects. Enhancement factors were calculated from three independent batches (Supplementary Table S6), with standard deviations below 5%, confirming reproducibility. This pattern suggests enhanced efficacy of nanoemulsion formulation, potentially due to improved dispersion, stability, or bioavailability of the active compounds.

**Table 3:** Mortality percentage of cinnamon, clove, and peppermint essential oils and their formulated nanoemulsions against larvae of *L. serricorne* on tobacco leaves after 24, 48, and 72 h of exposure period.

Treatment	Conce (ppm)	Mortality (%) ± SE		
		24 h	48 h	72 h
Cinnamon NE	16	24.00 ± 1.53	34.00 ± 1.71	40.00 ± 1.74
	24	34.00 ± 1.71	42.00 ± 0.78	52.00 ± 1.32
	32	38.00 ± 1.44	45.00 ± 0.95	61.00 ± 1.36
	40	48.00 ± 1.44	50.00 ± 1.47	70.00 ± 1.62
Cinnamon oil	400	33.00 ± 1.16	37.00 ± 0.92	42.00 ± 0.75
	600	42.00 ± 0.68	48.00 ± 1.29	50.00 ± 0.95
	800	44.00 ± 0.58	50.00 ± 1.61	60.00 ± 0.71
	1000	51.00 ± 0.73	57.00 ± 0.93	63.00 ± 1.03
Clove NE	16	36.00 ± 1.49	46.00 ± 0.86	50.00 ± 1.95
	24	42.00 ± 0.78	58.00 ± 1.78	62.00 ± 1.30
	32	48.00 ± 0.99	67.00 ± 2.73	72.00 ± 1.90
	40	56.00 ± 1.54	70.00 ± 1.62	88.00 ± 0.93
Clove oil	400	39.00 ± 1.41	42.00 ± 0.86	53.00 ± 1.88
	600	49.00 ± 0.75	52.00 ± 1.34	56.00 ± 0.91
	800	52.00 ± 0.82	55.00 ± 2.27	78.00 ± 1.55
	1000	62.00 ± 1.54	71.00 ± 1.29	89.00 ± 0.71
Peppermint NE	16	30.00 ± 1.40	38.00 ± 1.14	46.00 ± 1.81
	24	38.00 ± 1.14	50.00 ± 1.83	72.00 ± 2.19
	32	54.00 ± 1.63	68.00 ± 1.95	78.00 ± 1.73
	40	66.00 ± 1.51	78.00 ± 1.73	90.00 ± 0.58
Peppermint oil	400	33.00 ± 0.98	41.00 ± 1.38	49.00 ± 1.20
	600	45.00 ± 2.03	56.00 ± 1.65	68.00 ± 1.87
	800	61.00 ± 1.83	72.00 ± 1.61	81.00 ± 1.28
	1000	70.00 ± 0.87	82.00 ± 1.18	92.00 ± 0.37
Cont	0.0	00.00±0.00	00.00 ± 0.00	00.00 ± 0.00

NE = Nanoemulsion. Values are presented as mean ± standard error (SE).

The comprehensive statistical analysis of essential oil formulations against *L. serricorne* mortality revealed distinct patterns in the relative importance of concentration, exposure time, and essential oil type (Table 4). Two-way ANOVA results demonstrated that concentration exerted the strongest and most consistent effects across all formulations and exposure times. For nanoemulsions, concentration effects were highly significant at 24 h (F (3,48) = 16.17, *p* < 0.001, η^2^ = 0.310), 48 h (F (3,48) = 14.74, *p* < 0.001, η^2^ = 0.290), and 72 h (F (3,48) = 15.65, *p* < 0.001, η^2^ = 0.303). Similarly, for pure essential oils, concentration showed highly significant effects at all time points (24 h: F (3,48) = 22.26, *p* < 0.001, η^2^ = 0.382; 48 h: F (3,48) = 19.08, *p* < 0.001, η^2^ = 0.346; 72 h: F (3,48) = 25.96, *p* < 0.001, η^2^ = 0.419). The large effect sizes (η^2^ = 0.29–0.42) indicate that concentration accounted for approximately 30–42% of the variance in mortality, establishing it as the primary determinant of efficacy. Essential oil type exhibited more limited and context-dependent effects. For nanoemulsions, oil type showed borderline significance at 24 h (F (2,48) = 2.96, *p* = 0.056, η^2^ = 0.052), significant at 48 h (F (2,48) = 3.54, *p* = 0.032, η^2^ = 0.062), and borderline significance at 72 h (F (2,48) = 2.74, *p* = 0.069, η^2^ = 0.048). For pure essential oils, similar patterns emerged with borderline significance at 24 h (F (2,48) = 2.92, *p* = 0.058, η^2^ = 0.051) and significance at 72 h (F (2,48) = 3.74, *p* = 0.027, η^2^ = 0.065), but non-significance at 48 h (F (2,48) = 1.72, *p* = 0.184, η^2^ = 0.031). The smaller effect sizes (η^2^ = 0.03–0.07) indicate that oil type accounted for only 3–7% of mortality variance.

No significant two-way interactions were detected between oil type and concentration for either formulation at any exposure time (Table 4). For nanoemulsions, Oil Type × Concentration interactions were non-significant at 24 h (F (6,48) = 0.52, *p* = 0.790, η^2^ = 0.028), 48 h (F (6,48) = 0.25, *p* = 0.957, η^2^ = 0.014), and 72 h (F (6,48) = 0.16, *p* = 0.987, η^2^ = 0.009). For pure essential oils, interactions were similarly non-significant at 24 h (F (6,48) = 1.21, *p* = 0.306, η^2^ = 0.063), 48 h (F (6,48) = 0.28, *p* = 0.944, η^2^ = 0.016), and 72 h (F (6,48) = 0.61, *p* = 0.722, η^2^ = 0.033). The negligible effect sizes (η^2^ = 0.01–0.06) and consistent non-significance across all analyses indicate that the effects of oil type and concentration are independent and additive rather than interactive.

**Table 4:** Two-way ANOVA results for the effects of essential oil type and concentration on mortality of *L. serricorne* exposed to nanoemulsions at different exposure times.

Analysis	Factors	Nanoemulsions (16–40 ppm)			Pure essential oils (400–1000 ppm)		
		24 h	48 h	72 h	24 h	48 h	72 h
Two-Way Anova	Oil Type	F = 2.96	F = 3.54*	F = 2.74	F = 2.92	F = 1.72	F = 3.74*
		*p* = 0.056	*p* = 0.032	*p* = 0.069	*p* = 0.058	*p* = 0.184	*p* = 0.027
		η^2^=0.052	η^2^=0.062	η^2^=0.048	η^2^=0.051	η^2^=0.031	η^2^=0.065
	Concentration	F = 16.17***	F = 14.74***	F = 15.65***	F = 22.26***	F = 19.08***	F = 25.96***
		*p* < 0.001	*p* < 0.001	*p* < 0.001	*p* < 0.001	*p* < 0.001	*p* < 0.001
		η^2^=0.310	η^2^=0.290	η^2^=0.303	η^2^=0.382	η^2^=0.346	η^2^=0.419
	Interaction	F = 0.52	F = 0.25	F = 0.16	F = 1.21	F = 0.28	F = 0.61
		*p* = 0.790	*p* = 0.957	*p* = 0.987	*p* = 0.306	*p* = 0.944	*p* = 0.722
		η^2^=0.028	η^2^=0.014	η^2^=0.009	η^2^=0.063	η^2^=0.016	η^2^=0.033

η^2^ = partial eta squared (effect size: 0.01 = small, 0.06 = medium, 0.14 = large); Significance levels, * *p* < 0.05, *** *p* < 0.001; F = F-values and p = p-values.

Three-way analysis of variance (ANOVA) using life stage (adult vs. larva), essential oil (EO) type, and concentration as fixed factors were performed during this analysis (Table 5). The results confirmed that the major effect of life stage on mortality was not significant (*P* > 0.05). Furthermore, no significant interactions were discovered between life stage and EO type (*P* > 0.05), life stage and concentration (*P* > 0.05), or among all three factors (*P* > 0.05), based on mortality responses of adults and larvae were statistically the same across EO types and concentrations, data from both life stages were combined to simplify data presentation and improve clarity in Table 5.

Post-hoc Tukey HSD tests revealed specific patterns within significant main effects (Table 5). For nanoemulsions at 48 h, where oil type showed significant effects, clove oil produced significantly higher mortality than peppermint oil (mean difference = 1.98%, *p* = 0.032), while cinnamon oil showed intermediate mortality not significantly different from either. For pure essential oils at 72 h, both cinnamon and clove oils produced significantly higher mortality than peppermint oil (mean differences: 1.63%, *p* = 0.049), with no significant difference between cinnamon and clove oils. Concentration effects followed clear dose-response relationships for both formulations. For nanoemulsions, the highest concentration (40 ppm) produced significantly lower mortality than all lower concentrations across all time points (mean differences: 5.13–6.27%, all *p* < 0.001). Similarly, for pure essential oils, the highest concentration (1000 ppm) resulted in significantly higher mortality than all lower concentrations (mean differences: 4.74–6.48%, all *p* < 0.001). These patterns confirm strong linear relationships between concentration and mortality for both formulations.

**Table 5:** Multiple comparisons (Tukey HSD) for significant effects.

Analysis	Factors	Comparison	%MD	SE	*p*-value	95% CI
Nanoemulsion	48 h: Oil Type	Clove - Peppermint	1.975	0.775	0.032*	0.134, 3.816
		Cinnamon - Peppermint	1.500	0.775	0.134	−0.341, 3.341
		Cinnamon - Clove	−0.475	0.775	0.813	−2.316, 1.366
Essential oil	72 h: Oil Type	Cinnamon - Peppermint	1.612	0.685	0.053	−0.016, 3.241
		Clove - Peppermint	1.632	0.685	0.049*	0.004, 3.261
		Cinnamon - Clove	−0.020	0.685	1.000	−1.648, 1.608
Concentration	(Nano 24 h)	16ppm − 40ppm	5.133	0.768	< 0.001***	3.129, 7.137
		16ppm − 32ppm	3.333	0.768	< 0.001***	1.329, 5.337
		24ppm − 40ppm	3.300	0.768	< 0.001***	1.296, 5.304
Concentration	(EO 72 h)	400ppm − 1000ppm	6.483	0.791	< 0.001***	4.419, 8.548
		400ppm − 800ppm	4.223	0.791	< 0.001***	2.159, 6.288
		600ppm − 1000ppm	4.817	0.791	< 0.001***	2.752, 6.881

SE = Standard Error; CI = Confidence Interval; *p < 0.05, ***p < 0.001; NS = Not Significant (*p* > 0.05); η^2^ = partial eta squared (effect size): 0.01 = small, 0.06 = medium, 0.14 = large; MD = Mean Difference in mortality percentage; Values shown as ranges represent minimum-maximum across time points; All F-values and p-values are from Type III Sum of Squares; Repeated measures ANOVA includes both adults and larvae results.

Repeated measures ANOVA confirmed that mortality significantly increased with exposure time for both formulations and life stages (Table 6). For nanoemulsions, time effects were highly significant for adults (F (2,96) = 34.80, *p* < 0.001, η^2^ = 0.420) and larvae (F (2,96) = 26.79, *p* < 0.001, η^2^ = 0.358). For pure essential oils, similar strong time effects were observed for adults (F (2,96) = 36.38, *p* < 0.001, η^2^ = 0.431) and larvae (F (2,96) = 29.19, *p* < 0.001, η^2^ = 0.378). The large effect sizes (η^2^ = 0.36–0.43) indicate that exposure time accounted for 36–43% of mortality variance. Critically, Time × Oil Type and Time × Concentration interactions were non-significant for all analyses (all *p* > 0.05), indicating that temporal mortality patterns were consistent across different oils and concentrations.

**Table 6:** Repeated measures ANOVA results for the effects of exposure time on mortality of *L. serricorne*.

Analysis	Treatment	Factors	Life stage	η^2^	*p*	F	df
Repeated measures	Nanoemulsions (16–40 ppm)	Time effect	Adults	0.420	< 0.001	34.80	2,96
			Larvae	0.358	< 0.001	26.79	2,96
		Time×oil	Adults	0.011	0.881	0.293	4,96
			Larvae	0.011	0.941	0.191	4,96
		Time×conc	Adults	0.003	0.982	0.180	6,96
			Larvae	0.016	0.881	0.395	6,96
	Pure essential oils (400–1000 ppm)	Time effect	Adults	0.431	< 0.001	36.37	2,96
			Larvae	0.378	< 0.001	29.18	2,96
		Time×oil	Adults	0.017	0.802	0.40	4,96
			Larvae	0.007	0.951	0.17	4,96
		Time×conc	Adults	0.051	0.524	0.86	6,96
			Larvae	0.070	0.312	1.20	6,96

η^2^ = partial eta squared (effect size: 0.01 = small, 0.06 = medium, 0.14 = large); Significance levels: ns = not significant (*p* > 0.05), *p* < 0.001; *F* = F-values. *P* = p-values.

In general, the statistical analysis establishes a clear hierarchy in the determinants of essential oil efficacy against *L. serricorne*. Concentration emerged as the most important factor, with consistently large effect sizes and extreme statistical significance across all analyses. Exposure time ranked second in importance, also showing large effect sizes and high significance. Essential oil type represented a tertiary factor, showing smaller, less consistent effects that were significant only at specific time points. The complete absence of significant interactions further emphasizes the independent and additive nature of these treatment effects.

### Fumigant toxicity

The concentration-mortality responses for cinnamon, clove, and peppermint essential oils and their nanoemulsion (NE) formulations against adult and larval stages were successfully modeled using probit analysis. All 12 models showed adequate goodness-of-fit, as confirmed by non-significant Pearson chi-square tests (*p* > 0.05), validating the reliability of the estimated lethal concentration parameters for comparison (Table 7). The probit analysis uncovered notable differences in lethal concentration estimates across formulations, botanical compounds, and insect developmental stages. Nanoemulsion formulations consistently exhibited significantly higher toxicity compared to their respective pure essential oils. The median lethal concentration (LC₅₀) values ranged from 0.046 ppm for cinnamon and peppermint NEs against adults to 133.614 ppm for peppermint oil against larvae, reflecting a potency difference of nearly four orders of magnitude. However, all vapor-phase concentrations should be interpreted as nominal due to propablee potential losses to adsorption. Statistical comparisons of lethal concentrations were performed using the criterion of non-overlapping 95% confidence intervals, which indicates statistically significant differences at α = 0.05.

### Enhancement of toxicity *via* nanoemulsification

The nanoemulsion formulation dramatically enhanced the insecticidal potency of all three essential oils. Enhancement factors were calculated from mean LC₅₀s of three independent NE batches; standard deviations are reported in Table S6. Inter-batch variability was minimal (< 5%), confirming reproducibility. This enhancement was quantified using the Enhancement Factor (EF), calculated as the ratio of the LC_50_ of the pure oil to the LC_50_ of its corresponding nanoemulsion (Table 7). The nanoemulsions were 17.1 to 143.5 times more toxic than the pure oils against adults, and 31.5 to 77.1 times more toxic against larvae. The most substantial enhancement was observed for peppermint nanoemulsion against adults (EF = 143.5).

For every botanical compound and life stage combination, the 95% confidence intervals for the LC₅₀ values of nanoemulsions were completely separated from those of their corresponding pure oils. This consistent pattern of non-overlap provides definitive statistical evidence that nanoemulsion formulations possess significantly enhanced insecticidal activity. The calculated enhancement factors ranged from 17.1 (clove, adults) to 143.5 (peppermint, adults), with the greatest magnitude of enhancement observed for peppermint nanoemulsion against adult stages (Table 7).

**Fig. 5:**
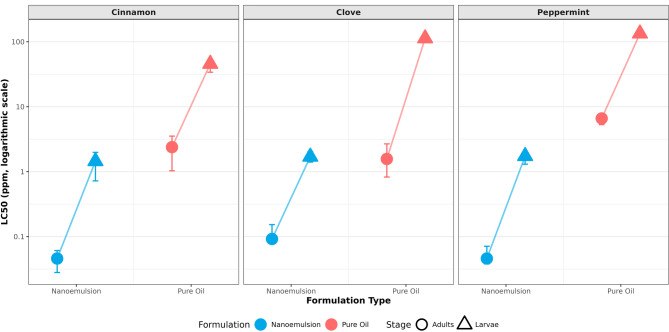
Paired comparison of LC_50_ values across essential oil formulations and developmental stages. Points represent mean LC_50_ estimates with 95% confidence intervals (error bars). Connected dashed lines illustrate the susceptibility gap between adult and larval stages within each formulation type. Lower LC_50_ values indicate higher toxicity. The logarithmic scale accommodates the wide range of toxicity values. NE = nanoemulsion formulation. Non-overlapping confidence intervals between adult and larval values indicate statistically significant differences (α = 0.05).

For every treatment (essential oils and formulation), the confidence intervals for LC₅₀ estimates in adults and larvae were entirely distinct, demonstrating no statistical overlap (Table 7; Fig. 5). The LC₅₀ for larvae exceeded that of adults by a factor of approximately 10 for cinnamon NE and more than 20 for peppermint oil. These non-overlapping confidence intervals confirm that adult stages exhibit significantly greater susceptibility to all formulations compared to their larval counterparts.

The slope parameters of the probit models, representing the steepness of the concentration-response relationship, exhibited consistent patterns. Nanoemulsions generally produced steeper slopes (408–1476 × 10^−3^) compared to pure oils (4–99 × 10^−3^), indicating more predictable concentration-dependent mortality. Within each treatment, adult stages consistently demonstrated steeper slopes than larval stages, reflecting their greater physiological sensitivity to concentration increments.

**Table 7:** Fumigant toxicity and enhancement factors for cinnamon, clove, peppermint free EO and its formulated nanoemulsions against the adults and larvae of *L. serricorne* after 72 h of exposure period.

Treatment	Stage	LC50 (ppm) [95% CI]	LC95 (ppm) [95% CI]	Slope ± SE (×10^−3^)	χ^2^ (*p*-value)	EF
Cinnamon NE	Adults	0.046 [0.028–0.061)	1.216 [0.960–1.363]	1303 ± 250	0.634 (0.911)	51.9
	Larvae	1.448 [0.721–1.984]	5.476 [4.666–7.003]	408 ± 77	0.709 (0.688)	31.5
Cinnamon oil	Adults	2.387 [1.032–3.526]	25.321 [23.232–30.295]	72 ± 10	0.617 (0.966)	–
	Larvae	45.633 [33.968–50.631]	177.998 [167.336–183.184.336.184]	12 ± 3	0.678 (0.777)	
Clove NE	Adults	0.092 [0.077–0.153]	1.207 [0.979–1.646]	1476 ± 247	0.902 (0.206)	17.1
	Larvae	1.696 [1.405–2.048]	5.266 [4.571–6.604]	461 ± 78	0.517 (1.318)	66.4
Clove oil	Adults	1.569 [0.827–2.679]	18.208 [15.569–22.355]	99 ± 13	0.485 (1.446)	–
	Larvae	112.672 [111.421–113.379.421.379]	537.769 [461.241–613.379.241.379]	4 ± 3	0.910 (0.188)	
Peppermint NE	Adults	0.046 [0.038–0.071]	1.216 [0.860–2.063]	1303 ± 250	0.634 (0.911)	143.5
	Larvae	1.734 [1.303–2.020]	4.568 [3.094–5.367]	580 ± 82	0.212 (3.099)	77.1
Peppermint oil	Adults	6.603 [5.302–7.843]	68.301 [55.526–71.008]	27 ± 9	0.304 (2.384)	–
	Larvae	133.614 [125.304–144.806.304.806]	355.023 [235.410–441.358.410.358]	7 ± 3	0.987 (0.026)	

CI = Confidence Interval. Slope values (×10^−3^) represent the PROBIT parameter estimate (B) from SPSS output multiplied by 1000. The corresponding standard error (SE) for each slope has also been multiplied by 1,000. χ^2^ represents the Pearson goodness-of-fit statistic with df = 2. All models showed adequate fit (*p* > 0.05). All models showed a good fit to the Probit model (*p* > 0.05 for Chi-Square GOF test). EF: Enhancement Factor = (LC₅₀ of Oil/LC₅₀ of NE). Significant differences in LC₅₀ values were inferred based on non-overlap of 95% confidence intervals (CIs).

## Discussion

Recently, there has been growing interest in EO-based biopesticides due to their ability to reduce the harmful effects of chemical pesticides and provide environmentally friendly alternatives^77^. Essential oils (Eos) are volatile and quite unstable, readily oxidizing under room conditions. Therefore, to ensure stability and protection, a nanoemulsion approach can be utilized as a promising methodology to augment the bioavailability and efficacy of compounds capable of enhancing the absorption and effectiveness of diverse active substances. There is a dearth of research assessing the effectiveness and implications of nanoemulsion treatments on stored tobacco insects. The comprehensive characterization of the fabricated peppermint, clove, and cinnamon nanoemulsions (NEs) confirms the successful formation of stable, nanoscale colloidal systems with distinct physicochemical properties. Transmission electron microscopy (TEM) analysis provided direct visual evidence of the morphological characteristics of nanoemulsions. The micrographs for all three formulations peppermint, clove, and cinnamon revealed spherical particles with a consistent morphology, which is a typical and desirable characteristic of oil-in-water nanoemulsions formed by high-energy emulsification methods^45,78–80^. The absence of irregular aggregates and the uniform dispersion observed under TEM suggest effective emulsification and the stabilizing role of the surfactant used in the formulation.

The particle size analysis, as determined from the log-normal fitting of the size distribution histograms, further substantiates the TEM observations. The data reveal a clear dichotomy in the sizes of the formulated NEs. The cinnamon NE demonstrated a remarkably small average diameter of 15.77 nm: significantly lower than that of both clove (44.81 nm) and peppermint (46.27 nm) NEs. This substantial difference can be attributed to the distinct chemical composition of the essential oils. Cinnamon oil, rich in cinnamaldehyde, may possess a greater affinity for the surfactant or contribute to a lower interfacial tension, facilitating the formation of finer droplets during homogenization. In contrast, the similarity in droplet sizes between clove (dominated by eugenol) and peppermint (abundant in menthol) NEs implies comparable physicochemical behavior at the oil-water interface in these emulsions.

The connection between physicochemical characteristics and enhanced functionality is central to rational nanomaterial design. Xu et al^81^. provide a compelling illustration of this principle, showing how the engineered “nano-gap” architecture of their 3D nanohelices enabled preferential water transport, resulting in a twofold increase in membrane permeance. Analogously, the improved efficacy of our clove NE can be traced to its specific physicochemical features: an exceptionally high zeta potential (−50.5 mV) ensures strong electrostatic stabilization, while its nanoscale droplet size (44.81 nm) maximizes surface area interaction with the insect cuticle. This synergy between structure and function underscores the effectiveness of our formulation approach.

The chemical composition of each oil (GC–MS, Supplementary Table S4) informs observed bioactivity, e.g., the high eugenol content in clove oil correlates with its potent insecticidal effects, supported by previous literatures. Based on the GC–MS profiles consolidated (Supplementary Table S4), cinnamon essential oil is predominantly composed of trans-cinnamaldehyde (66.3–81.9%), with eugenol (5–18%) and linalool (1–5%) as notable secondary constituents^82–86^. Clove oil is characterized by a high proportion of eugenol (~ 77–90%), accompanied by eugenyl acetate (~ 1.2–11.5%) and β-caryophyllene (trace–17.4%) as secondary components^87–89^. In peppermint oil, menthol constitutes the major component (41.2%), with menthone (23.8%) and 1,8-cineole (6.9%) providing significant secondary fractions^90^. These compositional profiles, consistently reported in the cited literature, underpin both the physicochemical properties and the bioactivity of the oils, reinforcing their relevance in the context of botanical insecticide development.

The comparable sizes of clove (eugenol-rich) and peppermint (menthol-rich) NEs suggest similar physicochemical interactions at the oil-water interface in these systems. Crucially, all formulations exhibited excellent monodispersity, as indicated by the low standard deviations (~ 1.2 nm) and exceptionally low coefficients of variation (CV < 7.5%). A CV value below 10% is generally considered indicative of a narrow size distribution and a homogeneous colloidal system^91^. The low polydispersity is a critical factor for the physical stability of nanoemulsions, as it minimizes the rate of Ostwald ripening a common destabilization mechanism where smaller droplets dissolve and redeposit onto larger ones.

The stability of the nanoemulsions was quantitatively assessed through zeta potential measurements. According to established colloid science principles, zeta potential values exceeding ± 30 mV are indicative of stable systems due to strong electrostatic repulsion that prevents droplet coalescence^92^. In this study, the highly negative zeta potential values observed, especially for clove NE (−50.5 mV), are indicative of strong electrostatic repulsion between droplets, which is a primary mechanism for ensuring long-term kinetic stability by preventing aggregation and coalescence. This finding is consistent with emerging principles in emulsion science, where ultralow concentrations of surface-active agents can achieve stable formulations through cooperative electrostatic interactions^59^. The magnitude of the zeta potential in our formulations, particularly for clove NE, suggests a robust electrostatic barrier, explaining the excellent monodispersity and lack of aggregation observed in TEM micrographs. The slightly acidic pH values (ranging from 4.50 for cinnamon to 5.60 for clove) in agreement with the results of prior work^52–70^ are consistent with the composition of the essential oils and are not expected to adversely affect the formulation’s stability, as evidenced by the high zeta potentials. Stability conclusions are limited to laboratory physicochemical indices (pH, zeta potential, DLS size) and do not encompass performance under real storage/usage conditions. Further work is required to assess stability under fluctuating temperature, humidity, and light over extended periods as well as in field or industrial conditions.

An interesting correlation can be drawn between the particle size and zeta potential. While the cinnamon NE possessed the smallest particle size, its long-term stability against aggregation may be challenged by its lower absolute zeta potential compared to clove NE. Conversely, the clove NE, with its larger droplet size but superior zeta potential, is predicted to have exceptional long-term colloidal stability. The peppermint NE presents an intermediate profile. This interplay between size and surface charge is vital for forecasting the shelf-life and functional performance of these nanoemulsions.

The application of probit analysis in the contact toxicity assay provided robust and statistically valid estimates of the lethal concentrations (LC₅₀ and LC₉₅) for essential oil nanoemulsions (NEs) and their pure oil counterparts against both adult and larval stages of *L. serricorne*. The results unequivocally demonstrate that nanoemulsification serves as a powerful formulation strategy to drastically enhance the insecticidal efficacy of essential oils, while also revealing critical differences in toxicity profiles and life-stage susceptibility. The most significant finding is the profound enhancement of toxicity achieved through nanoemulsification for every tested oil, the nanoemulsion was significantly more potent than its unformulated counterpart, as confirmed by non-overlapping 95% confidence intervals (CIs) at the LC₅₀ level. The magnitude of this enhancement, quantified by the enhancement factor (EF), was substantial, ranging from 9.4 to over 162-fold. Furthermore, the steep slopes of the probit regression lines for NEs indicate a more consistent and quick toxic effect across the test population as concentration increases. Steeper probit slopes in NE treatments suggest more homogeneous population responses, possibly due to uniform distribution and rapid absorption of actives. Conversely, shallow slopes for pure oils may reflect heterogeneous exposure or variable penetration. Conversely, the very shallow slopes for pure oils suggest poor and inconsistent delivery of the active ingredients, resulting in a varied and gradual mortality response. This dramatic increase in potency aligns with established principles of nano-formulation in pesticide science. Our result corroborated with the previous finding of Ousama et al.^93^, demonstrated that a nanoemulsion of *Cymbopogon winterianus* essential oil was more effective against *Tribolium castaneum* larvae and adults than the oil alone. Similarly, an anise nanoemulsion proved to be the most toxic option against *Sitophilus oryzae* and *T. castaneum*, reportedly 1.41–1.48 times more toxic than the bulk essential oil^94^. Further supporting this, a study on *Mentha piperita* essential oil found its nanoemulsion exhibited higher contact toxicity against adult *T. castaneum* after 72 h^95^. The variation in toxicity among different nanoemulsions is likely attributable to differences in the composition and relative abundance of their active components^96^.

The study revealed important nuances in toxicity among the different nanoemulsions. Against adult insects, clove NE emerged as the most potent adulticide, exhibiting significantly higher toxicity than cinnamon or peppermint NEs. For larval control, clove NE was unequivocally the superior larvicide, with an LC₅₀ an order of magnitude lower than the other NEs. This suggests that the bioactive compounds in clove oil (primarily eugenol) are not only highly toxic to this insect species but are also particularly amenable to delivery via the nanoemulsion system, resulting in synergistic efficacy. Similar results are in close conformity with the finding of Ikawati et al.^34^, who found that clove oil nanoparticles were less LC values to *T. castaneum* than free clove oil in terms of residual contact toxicity. El Gohary^97^ studied the LC values of clove EO and clove chitosan-NPs on *Culex pipiens* third instar larvae and discovered that treated larvae were more vulnerable to clove -chitosan-NPs than clove EO. Based on the LC_50_, the comparable potency of cinnamon and peppermint NEs against adults, and their lower potency against larvae compared to clove NE, highlights that the success of a nano-formulation is contingent upon both the chemical properties of the specific essential oil and the target life stage^98^.

The analysis of stage-specific susceptibility yielded another critical insight: nanoemulsification can alter the inherent susceptibility patterns observed with pure oils. For pure oils, susceptibility did not differ significantly between adults and larvae. However, when formulated as NEs, both cinnamon and clove became significantly more toxic to larvae than to adults. This differential effect is best explained by the interplay between the insect’s biology, which may also be rooted in underlying physiological and metabolic differences. and the unique action of nanoemulsions. The greater susceptibility of larvae may be due to their thinner, less sclerotized cuticle, higher metabolic rates, or greater surface-to-volume ratio, facilitating increased penetration and efficacy of nanoemulsions. Future studies should include cuticular penetration assays and enzymatic activity measurements. Research on another coleopteran, Tenebrio molitor, has shown that insect lipid metabolism is highly dynamic and can be significantly altered by dietary components, leading to the incorporation of fatty acids into complex phospholipids^65^. This suggests that the enhanced efficacy of nanoemulsions might involve more efficient integration into, or disruption of, the insect’s critical metabolic pathways, such as glycerophospholipid metabolism, once the active compounds have penetrated the cuticle. The softer, developing circle of larvae, combined with their high metabolic rate for growth, could make them particularly susceptible to such metabolic interference by bioactive compounds like those in essential oils. Physiologically, larvae are inherently more vulnerable. Their soft, less sclerotized cuticle, essential for growth and molting^74^, offers little resistance to Nanoemulsion. The formulation’s surfactants and nano-scale droplets (20–200 nm) enhance cuticular penetration by disrupting epicuticular lipids and providing a large surface area for interaction. While adults are protected by hardened exoskeleton^39^, this mechanism proves catastrophic for larvae, leading to systemic toxin entry. Behaviorally, larval ecology maximizes their exposure. Their cryptic, sedentary life within the food substrate ensures prolonged contact with treated surfaces during feeding^18^. In contrast, mobile adults have intermittent contact. Therefore, the contact bioassay directly targets the larval life stage within its primary habitat^90,99^. The superior efficacy of the nanoemulsion against larvae is thus a product of both the formulation’s enhanced bioavailability and the specific biological and behavioral vulnerabilities of this critical life stage. The exception was peppermint NE, to which both stages remained equally susceptible. This differential effect underscores that the interaction between the nano-vehicle and the insect’s biology is complex and oil-dependent.

In insecticidal efficacy the assay with treated tobacco leaves is crucial for controlling *L. serricorne*, a pest that significantly damages stored tobacco leaves depending on EO type and formulation as well as their concentration and exposure duration. Our findings revealed varied levels of insecticidal activity against *L. serricorne* larvae and adults. This study successfully demonstrates the insecticidal efficacy of cinnamon, clove, and peppermint essential oils (EOs) and their nanoemulsion (NE) formulations against both adult and larval stages of *L. serricorne*. While direct statistical comparison between nanoemulsions and pure essential oils was not conducted due to different concentration ranges, descriptive comparisons reveal interesting patterns. Nanoemulsions achieved substantial mortality at much lower concentrations (16–40 ppm) compared to pure oils (400–1000 ppm), suggesting enhanced efficacy through improved dispersion and bioavailability. This is consistent with the nanotechnology principle that reducing particle size increases surface area and enhances biological activity^16^. The enhanced efficacy of nanoemulsions may be attributed to several factors: improved stability of active compounds, enhanced penetration through insect cuticles, reduced volatility, and better distribution on treated surfaces. These advantages align with the growing body of literature supporting nanotechnology applications in pest management.

The repeated measures ANOVA unequivocally confirmed that concentration optimization and adequate exposure duration are the primary determinants of essential oil efficacy against *L. serricorne*, while essential oil selection plays a secondary role. These findings redirect focus from identifying “superior” oils toward optimizing application parameters, but in the innovative formulation and delivery of known active oils to achieve and maintain biologically critical concentration at the target site. This temporal pattern is biologically plausible, as longer exposure durations allow for greater absorption, distribution, and action of the active compounds on target sites^100^. The essential oils tested in this investigation were chosen based on their recognised insecticidal effectiveness. Even though other essential oils may also display high bioactivity, curent results recommend that, once effective oils are identified, the formulation strategy and request method play a more significant role in determining insecticidal efficacy than the specific essential oil used. This strong dose-response relationship aligns with fundamental toxicological principles, where increased toxicant concentration typically enhances biological effects through greater target site occupancy and reduced detoxification capacity. The absence of significant Time × Treatment interactions indicates that all formulations and concentrations followed similar temporal mortality trajectories. These results aligned with the work of^70^, who found that mortality of *S. oryzae* increased progressively from 24 to 72 h when exposed to peppermint and garlic essential oils. The consistency of time effects for different formulations suggests that the rate-limiting steps in toxicity (absorption, metabolic activation, or target site interaction) operate similarly regardless of formulation type. This has practical implications for pest management, indicating that even sub-lethal exposures over extended periods can achieve effective control^101^. Also, Stejskal et al^102^. similarly demonstrated that increasing concentrations of thyme and oregano essential oils produced progressively higher mortality in *L. serricorne*, with mortality rising from 15% to 95% as concentration increased from 0.5 to 5.0 µL/L air. The mechanistic basis for this concentration dependence likely involves multiple factors: higher concentrations increase the probability of contact with target sites, enhances penetration through the insect cuticle, saturates metabolic detoxification systems, and potentially overwhelms physiological compensation mechanisms^103^.

The enhanced efficacy we observed in nanoemulsion formulations aligns with findings from other stored-product pest systems. For instance, nanoemulsions have been shown to increase the mortality of *T. castaneum* and *S. oryzae*compared to pure oils, Iqbal et al^63^. who found that nanoemulsions containing clove EO have demonstrated promising results in controlling *T. castaneum* adults and larvae, further supporting the efficacy of EO nanoemulsions as pest control agents. After seven days, exposure to A 1000 mg/L geranium essential oil nanoemulsion caused 100%, 70%, and 100% death in *T. castaneum*,* S. granarius*, and *Oryzaephilus surinamensis*, respectively. In both male and female adults of *O. surinamensis*, Anas et al. and Gharsan^52,104^ found that nanoemulsions showed higher adult mortality than pure oil. By using the thin film residue technique, Massoud et al.^39^ investigated the impact of *M. pipe*rita essential oil nanoemulsion on *S. oryzae* and demonstrated that the *M. piperita* (4%) nanoemulsion had the strongest and most rapid toxic effect. Similarly, turmeric nanoemulsions showed strong insecticidal activity against *S. oryzae*^58^, followed by frankincense and sesame nanoemulsions. Furthermore, castor nanoemulsion was the most toxic to *S. oryzae*, *T. castaneum*, and *O. surinamensis*^70,79,105^. The effectiveness of pure citronella essential oil more than doubled when formulated into a nanoemulsion, according to^92^.

The most striking result for fumigant action is the consistent and statistically significant superiority of NE formulations over their pure EO counterparts. The definitive lack of overlap in the 95% confidence intervals for LC₅₀ values between NEs and pure oils for all botanical compounds and life stages provides conclusive evidence of enhanced efficacy. LC₅₀ comparisons relied on both CI overlap and likelihood ratio tests (Supplementary Table S5), strengthening statistical claims. LC₅₀ comparisons relied on both CI overlap and likelihood ratio tests (Supplementary Table S5), strengthening statistical claims. The magnitude of enhancement, quantified by factors ranging from 17.1 to 143.5, is substantial. In line with our results, studies have shown that essential oil nanoemulsions were significantly more toxic and effective as fumigants than their bulk forms. Arslan Azam et al.^106^, who found that *Eucalyptus microtheca* and *Eucalyptus rudis* NEs outperformed their EOs in terms of fumigation toxicity. Also, Ibrahim et al.^107^ showed that geranium essential oil is an efficient fumigant against *Callosobruchus maculatus*, with the nanoemulsion formulation having greater and longer-lasting insecticidal action than the bulk oil while using significantly less active material. In the same way, several essential oil nanoemulsions have proved to be more efficient against agriculture and stored food pests than the free essential oil, which is appreciated in a lower concentration to neutralize 50% (LC_50_) in fumigant tests^42,108^. When compared to the effects of the free monoterpene, the fumigant application of 1,8-cineole nanoemulsion formulation showed higher acute toxicity against adult two-spotted spider mites, corn aphids, and silverleaf whiteflies, lowering the LC_50_ parameter by more than 50% for all three pests. In a related investigation, *Myristica fragrans* and *Jatropha curcas* nanoemulsion formulations showed toxicity to adult *S. zeamais* through seed dressing, contact, and fumigation activities^109^. In another study, the nanoemulsion made from sweet orange (*Citrus sinensis*) essential oil formulation was shown to be acutely toxic to the confused flour beetle and flat grain beetle when evaluated as a fumigant^110^. Also, on the larvae and eggs of the Mediterranean flour moth, Louni et al.^111^ showed that fumigant toxicity was maintained by a nanoemulsion containing the essential oil of Mentha longifolia with progressive release.

Numerous reasons pertaining to the special characteristics of NEs can be responsible for this increased effectiveness. Initially, nanoemulsions significantly improve the solubility of EOs in water^112^. This is accomplished by producing micelles that encapsulate hydrophobic EO molecules and facilitate their absorption via the insect body^113^. Second, the higher surface area of NE droplets provides for better interaction and contact between the NE and the insect cuticle, which may result in improved effectiveness^114^. Furthermore, the negative surface charge of NEs reduces particle agglomeration and increases their contact duration with the insect body^115^. These enhanced properties enable the volatile EO components of NEs to reach the insect’s respiratory, digestive, and cuticular systems more efficiently^116^.

 The differential efficacy of contact versus fumigant toxicity can be explained by formulation properties: volatility, viscosity, and surface tension affect mode-specific delivery and potency. Direct, quantitative benchmarking with previous studies is presented in Suplementary Table S7, demonstrating the superior or comparable efficacy of our NEs. Benchmarking the present study’s findings against prior research highlights the substantial advancement achieved with essential oil nanoemulsions (NEs) for stored product pest management. The clove NE exhibited a 23.4-fold enhancement in contact toxicity against adult *Lasioderma serricorne* (LC₅₀ = 220.42 ppm), a result broadly comparable to or better than those reported for *Tribolium castaneum* and *Sitophilus oryzae* using clove NE or nanocapsules, where enhancement factors and LC₅₀ values ranged from 2.2 to 40.0 and 45–175.5 ppm, respectively^58,62,63^. Peppermint NE in the present study (LC₅₀ = 289.16 ppm, EF = 9.4) demonstrated higher efficacy than comparable peppermint and geranium NEs tested against *S. oryzae* or *T. castaneum*^58,67^. While some studies utilizing different oils or encapsulation matrices (e.g., castor NE, geranium NE, citronella NE) reported varying enhancement factors and LC₅₀ values, the current work underscores consistent and substantial improvements in potency, often at markedly lower concentrations than those required in earlier formulations^1,80,114^. Collectively, these results establish the superior efficacy and broad applicability of essential oil NEs, particularly clove and peppermint, for sustainable pest control in stored tobacco and other commodities.

Nanoemulsification enhances toxicity through several mechanisms: (i) increased surface area and dispersion, (ii) improved cuticular penetration due to nanometric droplet size, (iii) reduced volatility leading to sustained exposure, and (iv) possible surfactant-mediated disruption of insect membranes^117^. The results demonstrate that nanoemulsification dramatically increased the insecticidal potency of essential oils, as shown by high enhancement factors (EFs) across all tested formulations. In contact toxicity assays, the greatest enhancement was observed for clove NE against larvae (EF = 162.1), while cinnamon and peppermint NEs also showed substantial increases, especially for larval stages. Similarly, for fumigation toxicity, peppermint NE achieved the highest EF in adults (143.5), with all oils exhibiting at least a 17-fold increase in efficacy over their pure forms. Importantly, batch-to-batch variability in EF values was minimal, with standard deviations consistently below 5%, indicating high reproducibility and robustness of the nanoemulsion formulations. These findings confirm that nanoemulsification not only enhances the bioactivity of essential oils in both contact and fumigant applications but also delivers consistent performance across independent batches (Supplementary Table S6).

Our findings position clove, cinnamon, and peppermint nanoemulsions as particularly promising botanical insecticides for the control of *L. serricorne*. Their ability to induce high mortality at low concentrations addresses major limitations of pure EOs, namely their high volatility, poor water solubility, and need for large quantities. As demonstrated by^118^, metabolomic profiling can effectively map disrupted metabolic pathways and reveal the systemic effects of botanical treatments. Future research should focus on elucidating the precise mechanisms of action at physiological and molecular levels, evaluating the efficacy of these nanoformulations under real-world storage conditions, and assessing their safety for non-target organisms and human health to facilitate their integration into integrated pest management (IPM) protocols.

## Conclusion

This study elucidated the enhanced insecticidal efficacy and physicochemical characteristics of nanoemulsions (NEs) derived from cinnamon, clove, and peppermint essential oils. Physicochemical analysis confirmed the successful development of stable colloidal systems at the nanoscale. A key finding is the profound increase in efficacy against *L. serricorne*, with NEs consistently outperforming pure oils in both contact and fumigant bioassays. Contact toxicity was both formulation- and life stage-dependent, with clove NE emerging as the most potent candidate, particularly against larvae. Furthermore, the significantly steeper probit slopes of the NEs indicate a more predictable and rapid dose-response compared to the shallow slopes of the pure oils a desirable trait for practical applications. Notably, nanoemulsification introduced stage-specific efficacy: larvae showed heightened susceptibility to cinnamon and clove NEs in contact assays, while adults were markedly more vulnerable to all NEs in fumigation tests, with cinnamon and peppermint NEs being especially effective. On treated tobacco leaves, efficacy was governed primarily by concentration and exposure time, with oil type being of secondary importance. This study therefore establishes that the future of botanical insecticides lies not merely in discovering novel plant extracts, but also in advanced formulation strategies such as nanoemulsification that ensure delivery of critical bioactive concentrations to the target site. Utilizing GRAS ingredients, these NEs represent promising, sustainable alternatives to synthetic pesticides for sensitive settings, including post-harvest protection and urban pest management. While clove, cinnamon, and peppermint NEs showed strong potential as botanical insecticides for stored tobacco, Future work should now concentrate on three critical areas. First, the underlying mechanisms of action need to be determined. Second, the efficacy of these approaches must be validated through field trials that reflect real-world conditions. Finally, a thorough assessment is required to ensure no detrimental effects on tobacco quality and to confirm their safety for both non-target organisms and human health. Data on non-target effects, environmental fate, and impacts on tobacco quality remain to be generated.

## Supplementary Information

Below is the link to the electronic supplementary material.

Supplementary Material 1

## Data Availability

The datasets generated or analyzed during the current study are available on reasonable request.
