# Supplementary material for: Essential oil nanoemulsions enhance protection of stored tobacco against Lasioderma serricorne
Source: Sci Rep. 2026 Apr 9;16:12057. doi: 10.1038/s41598-026-45107-x (PMC13068949; doi:10.1038/s41598-026-45107-x)
Supplement: Supplementary file 1 — Supplementary Material 1 [file 41598_2026_45107_MOESM1_ESM.docx]

**Supplementary Methods**

**Method: converting volumes and masses into concentrations expressed in ppm**

This method describes the steps and formulas used to convert applied volumes of essential oil nanoemulsions (NEs) or pure oils into concentrations expressed in parts per million (ppm) for all toxicity bioassays (contact, treated tobacco leaves, and fumigation). This ensures that dosing is reproducible and transparent (supplementary methods). Depending on the assay, the substrate can be the surface area of a Petri dish (for contact), the mass of leaves (for treated tobacco), or the volume of air (for fumigation). The concentrations in ppm (parts per million) were estimated using the volume of the test agents administered and the capacity of the exposure chamber or treated surface area.

- 1. **For treated tobacco leaf assays (leaf-dipping method)**

**In case of nanoemulsion formulations**

The final concentration of the essential oil in parts per million (ppm) was determined as follows: we first calculated the amount of active oil present in each test volume of the 4% v/v product, which contains 0.04 µL of oil per 1 µL of product. Consequently, volumes of 10, 15, 20, and 25 µL of product contain 0.4, 0.6, 0.8, and 1.0 µL of active oil, respectively. The oil has a density of approximately 1 g/mL, which allows for a direct conversion of volume to mass. These volumes correspond to masses of 400, 600, 800, and 1000 µg of oil. Each of these masses is then diluted into 25 mL of acetone. Therefore, the final concentration in ppm (µg/mL) is calculated by dividing the mass of oil by the solvent volume, resulting in concentrations of 16, 24, 32, and 40 ppm for the 10, 15, 20, and 25 µL product volumes, respectively.

**In case of pure oil**

We calculated the concentration in parts per million (ppm) for each volume of essential oil added. We used the formula: Concentration (ppm) = (Volume of Oil in µL / Total Volume of Solution in mL) × 1000. Based on this formula, the concentrations for the experiment are as follows: Adding 10 µL of oil to 25 mL of solution yields 400 ppm; 15 µL in the same total volume gives 600 ppm (as seen in two instances); 20 µL results in 800 ppm; and 25 µL produces 1000 ppm.

- 1. **For contact toxicity assays (thin film method)**

**In case of nanoemulsion formulations**

We determine the concentration of active oil in a 2 mL acetone solution. Begin by converting the volume of product used from microliters to milliliters (e.g., 20 µL becomes 0.02 mL). Since the product contains 4% active oil by mass, multiply the product mass by 0.04 to find the mass of the active oil, which for 20 µL equals 0.0008 g. To express this in micrograms, multiply by 1,000,000, resulting in 800 µg. Finally, divide this mass by the final solution volume of 2 mL to obtain a concentration of 400 µg/mL, which is equivalent to 400 ppm, and 10 µL yields 200 ppm, 15 µL yields 300 ppm, and 25 µL yields 500 ppm.

**In case of pure oil**

The concentration of oil in each solution was determined by first calculating the mass of oil from its volume. The mass (in milligrams) was obtained using the formula mass = volume (µL) × density (g/mL). Because the volume of oil added, ranging from 10 to 25 µL, was negligible relative to the total solution volume, the overall volume was approximated as 2 mL. Concentration in mg/mL was then calculated by dividing the mass of oil by the solution volume. For instance, adding 10 µL of oil (with a mass of approximately 9 mg) to 2 mL of acetone yields a concentration of 4.5 mg/mL. Since 1 mg/mL is equivalent to 1000 ppm, this amount corresponds to 4,500 ppm of oil in acetone. The same method was applied to oil volumes of 15, 20, and 25 µL, resulting in concentrations of 6,750 ppm, 9,000 ppm, and 11,250 ppm, respectively.

- 1. **For fumigation assays in 250 mL jars**

**In case of nanoemulsion formulations**

We determined the nominal concentration of the active oil within the exposure chamber. The following calculations were performed based on the volume of product injected. The nanoemulsion product consisted of 4% (v/v) active oil. The volume of active oil introduced into the system was calculated using the following formula:

Volume of Active Oil (µL) = Volume of Product Injected (µL) × (4 / 100)

Concurrently, the internal volume of the exposure chamber (250 mL) was converted to liters to conform to the standard definition of parts per million (ppm) for vapor concentrations in air:

Chamber Volume (L) = 250 mL × (1 L / 1000 mL) = 0.250 L

The nominal concentration of the active oil vapor in the chamber atmosphere, expressed in parts per million, was subsequently calculated using the formula for gaseous mixtures, where ppm is defined as the volume of solute (in microliters) per volume of air (in liters):

Concentration (ppm) = (Volume of Active Oil in µL) / (Chamber Volume in L)

For an injection volume of 10 µL of the nanoemulsion product, the resulting concentration of the active oil blend in the 250 mL chamber was therefore calculated as follows:

(10 µL × 0.04) / 0.250 L = 1.6 ppm.

The same method was applied to oil volumes of 0.5, 1, 3, 5, 15, 20, and 25 µL, resulting in concentrations of 0.08, 0.16, 0.48, 0.80, 2.40, 3.20, and 4.00 ppm, respectively.

**In case of pure oil**

The oil concentration in each solution, expressed in parts per million (ppm), was determined in two steps. First, the oil mass (mg) was calculated using the formula of mass (mg) = volume (µL) × density (g/mL). The concentration was then obtained by dividing the calculated oil mass by the volume of the exposure chamber. With a chamber volume of 250 mL (0.250 L), the final concentration was computed as Concentration (ppm) = Mass of Oil (mg) / Volume of Chamber (L). The same method was applied to oil volumes of 0.5, 1, 3, 5, 10, 15, 20, and 25 µL, yielding concentrations of 2.1, 4.2, 12.6, 21.0, 42, 63, 84, and 105 ppm, respectively.

**Supplementary Tables**

**Supplementary Table S1.** Preliminary Surfactant Ratio Screening Results

| **Surfactant Ratio (Tween 80: Span 20)** | **Emulsion Clarity (1–5)*** | **Phase Separation at 7 Days (%)** | **Mean Droplet Size (nm, DLS)** | **PDI** |
| --- | --- | --- | --- | --- |
| 3:1 | 3 | 15 | 35.2 | 0.22 |
| 4:1 | 5 | 0 | 18.3 | 0.18 |
| 5:1 | 4 | 8 | 28.7 | 0.20 |

****Emulsion Clarity: 1=opaque, 5=clear.***

**Supplementary Table S2.** DLS Droplet Size and Polydispersity Index (PDI) of Nanoemulsions

| **Nanoemulsion** | **Mean Size (nm, DLS)** | **Standard Deviation (nm)** | **PDI** | **Mean Size (nm, TEM)** | **CV (%)** |
| --- | --- | --- | --- | --- | --- |
| **Cinnamon NE** | 18.3 | 2.1 | 0.18 | 15.77 | 7.4 |
| **Clove NE** | 48.2 | 3.8 | 0.19 | 44.81 | 2.5 |
| **Peppermint NE** | 51.7 | 4.2 | 0.17 | 46.27 | 2.6 |

**Supplementary Table S3.** Stability of Nanoemulsions Under Different Conditions

| **Condition** | **Cinnamon NE (nm, DLS)** | **Clove NE (nm, DLS)** | **Peppermint NE (nm, DLS)** | **Phase Separation Observed** |
| --- | --- | --- | --- | --- |
| **Initial (Day 0)** | 18.3 | 48.2 | 51.7 | No |
| **90 days @ 4°C** | 19.1 | 48.7 | 52.4 | No |
| **90 days @ 25°C** | 20.2 | 49.0 | 53.1 | No |
| **Freeze-thaw (3 cycles)** | 18.7 | 48.8 | 52.6 | No |
| **Centrifugation (10,000 rpm, 30 min)** | 18.6 | 48.5 | 51.9 | No |

**Supplementary Table S4.** GC–MS Profiles of Essential Oils (cited from previous studies)

| **Oil** | **Major Components** | **% Area** | **Secondary Components** | **% Area** | **Previous Study Citations** |
| --- | --- | --- | --- | --- | --- |
| **Cinnamon** | Trans-cinnamaldehyde | 66.3–81.9 | Eugenol | 5–18 | Mutlu et al. 2023;  Industrial Crops and Products, 2013;  ESCOP, 2003;  WHO summary, (1999 |
|  |  |  | Linalool | 1–5 |  |
|  | Cinnamaldehyde | 77.9 | Eugenol | 4.6 | Cantó Catalá & Blázquez Ferrer, 2017 |
|  |  |  | Linalool | 0.30 |  |
|  |  | 78.6 | Eugenol | 12.3 | Singh et al., 2007 |
|  |  |  | Linalool | 3.6 |  |
| **Clove** | Eugenol | ~80 | Eugenyl acetate | ~8 | Haro-González et al. (2021) |
|  |  |  | β-Caryophyllene | trace–2 |  |
|  |  | 89.9 | Eugenyl acetate | 7.9 | Fadel et al., 2020 |
|  |  |  | β‑Caryophyllene | 1.4 |  |
|  |  | 82.1 | Eugenyl acetate | 11.5 | Chaieb et al., 2007; |
|  |  |  | β-Caryophyllene | 2.1 |  |
|  |  | 76.8 | Eugenyl acetate | 1.2 | Jirovetz et al. (2006) |
|  |  |  | β‑Caryophyllene | 17.4 |  |
| **Peppermint** | Menthol | 41.2 | Menthone | 23.8 | Soković et al., 2009 |
|  |  |  | 1,8-Cineole | 6.9 |  |

**Supplementary Table S5.** Pairwise Statistical Comparisons (F-statistic tests) of LC₅₀ Values

| **NE vs Oil**  **Pairwise Comparison** | **LC₅₀ Values (ppm)** | **p-value** | **Significant**  **Difference?** |
| --- | --- | --- | --- |
| **Contact Toxicity:** |  |  |  |
| Clove NE vs. Clove Oil  (Adults) | 220.42 vs. 5147.75 | <0.001 | Yes |
| Clove NE vs. Clove Oil  (Larvae) | 32.71 vs. 5303.11 | <0.001 | Yes |
| Cinnamon NE vs. Cinnamon Oil  (Adults) | 299.45 vs. 6709.63 | <0.001 | Yes |
| Cinnamon NE vs. Cinnamon Oil  (Larvae) | 184.59 vs. 6878.32 | <0.001 | Yes |
| Peppermint NE vs. Peppermint Oil  (Adults) | 289.16 vs. 2704.34 | <0.001 | Yes |
| Peppermint NE vs. Peppermint Oil  (Larvae) | 275.62 vs. 12648.47 | <0.001 | Yes |
| **Fumigant Toxicity:** |  |  |  |
| Clove NE vs. Clove Oil  (Adults) | 0.092 vs. 1.569 | <0.001 | Yes |
| Clove NE vs. Clove Oil  (Larvae) | 1.696 vs. 112.672 | <0.001 | Yes |
| Cinnamon NE vs. Cinnamon Oil  (Adults) | 0.046 vs. 2.387 | <0.001 | Yes |
| Cinnamon NE vs. Cinnamon Oil  (Larvae) | 1.448 vs. 45.633 | <0.001 | Yes |
| Peppermint NE vs. Peppermint Oil  (Adults) | 0.046 vs. 6.603 | <0.001 | Yes |
| Peppermint NE vs. Peppermint Oil  (Larvae) | 1.734 vs. 133.614 | <0.001 | Yes |

**Supplementary Table S6.** Enhancement Factors and Batch Variability

| **Nanoemulsion** | **Life Stage/ oil formulation** | **Mean LC₅₀**  **(ppm)** | | **Enhancement**  **Factor (EF)** | **EF SD**  **(%)** |
| --- | --- | --- | --- | --- | --- |
|  |  | **NE** | **Oil** |  |  |
| **Based on mode of entry and action:** | | | | | |
| **Contact Toxicity:** | |  |  |  |  |
| Cinnamon NE | Adults | 299.45 | 6709.63 | 22.4 | 3.7 |
|  | Larvae | 184.59 | 6878.32 | 37.3 | 4.1 |
| Clove NE | Adults | 220.42 | 5147.75 | 23.4 | 3.5 |
|  | Larvae | 32.71 | 5303.11 | 162.1 | 4.4 |
| Peppermint NE | Adults | 289.16 | 2704.34 | 9.4 | 3.8 |
|  | Larvae | 275.62 | 12648.47 | 45.9 | 4.5 |
| **Fumigation Toxicity:** | |  |  |  |  |
| Cinnamon NE | Adults | 0.046 | 2.387 | 51.9 | 3.5 |
|  | Larvae | 1.448 | 45.633 | 31.5 | 3.9 |
| Clove NE | Adults | 0.092 | 1.569 | 17.1 | 3.2 |
|  | Larvae | 1.696 | 112.672 | 66.4 | 4.7 |
| Peppermint NE | Adults | 0.046 | 6.603 | 143.5 | 4.1 |
|  | Larvae | 1.734 | 133.614 | 77.1 | 5.0 |
| **Based on Oil type & Life Stage** | | | | | |
|  | | **EF (LC50)** | | **Interpretation** | |
| **Cinnamon** | Adults (NE vs. Oil) | 2.387 / 0.046 = 51.9 | | NE is ~52x more toxic than oil for adults. | |
|  | Larvae (NE vs. Oil) | 45.633 / 1.448 = 31.5 | | NE is ~32x more toxic than oil for larvae. | |
| **Clove** | Adults (NE vs. Oil) | 1.569 / 0.092 = 17.1 | | NE is ~17x more toxic than oil for adults. | |
|  | Larvae (NE vs. Oil) | 112.67 / 1.696 = 66.4 | | NE is ~66x more toxic than oil for larvae. | |
| **Peppermint** | Adults (NE vs. Oil) | 6.603 / 0.046 = 143.5 | | NE is ~144x more toxic than oil for adults. | |
|  | Larvae (NE vs. Oil) | 133.61 / 1.734 = 77.1 | | NE is ~77x more toxic than oil for larvae. | |

- **SD for LC₅₀ values**: Calculated based on the confidence intervals reported in Table 1 (contact) and Table 7 (fumigation) of the manuscript using the formula:
  **EF SD (%)**: Batch variability was below 5% for all EFs.

**Supplementary Table S7.** Benchmarking of Present Study Versus Previous Studies

| **Study/Citation** | **Pest Species** | **Oil/Formulation** | **Enhancement**  **Factor** | **Bioassay**  **Type** | **LC₅₀**  **(ppm)** | |
| --- | --- | --- | --- | --- | --- | --- |
|  |  |  |  |  | **Present**  **Study Value** | **Comparable**  **Literature Value** |
| **Present study** | L. serricorne  (Adult) | Clove NE | 23.4 | Contact | 220.42 | N/A |
| **Jasman et al. (2025)** | T. castaneum  (Adult) | Clove NE | 40.0 | Contact | 220.42 | 45 |
| Iqbal et al. (2025) | *T. castaneum* (Adult) | Clove NE | 2.2 | Contact | 220.42 | 132.7 |
| Abd El-Naby et al. (2023) | *T. castaneum* (Adult) | Castor NE | 8.5 | Contact | — | 485.8 |
| Nayak et al. (2025 | *S. oryzae* (Adult) | Clove Nano capsules | — | Contact | 220.42 | 175.5 |
| Abdul Qayyum et al. (2025) | *S. oryzae* / *T. castaneum* | Clove oil nanocapsules (PEG) | — | Residual contact | — | 2,260.89 / 10,498 mg/kg |
| **Abd El Salam et al. (2024)** | S. oryzae  (Adult) | Peppermint NE | 9.4 | Contact | 289.16 | 105 |
| Abd El‑Salam et al. (2024) | *S. oryzae* (Adult) | Geranium NE | — | Contact | 289.16 | 2.298 |
| Abd El‑Naby et al. (2024) | *R. dominica* (Adult) | Clove Nano capsules | — | Contact | 220.42 | 175.5 |
| Kavallieratos et al. (2023) | *S. oryzae* (Adult) | *Carlina acaulis* NE | — | Contact | — | 168.2 |
| Rohimatun et al. (2024) | *C. maculatus* (Adult) | Citronella NE | ~4.5 | Contact | — | 10030* |
| Heydari et al. (2020) | *Aphis gossypii* (Aphid) | Peppermint oil NE | — | Contact (aphicidal) | — | ~3,879.5 µl a.i./L |
| Lokesh et al. (2024) | *S. oryzae* (Adult) | Peppermint-piperitone NE | — | Fumigant | — | 30.65 |
| Narasimman et al. (2022) | *Anopheles stephensi* (Larvae) | Peppermint NE | — | Larvicidal (immersion) | — | 9.67 |
| Anjali et al. (2012) | *Culex quinquefasciatus* (Larvae) | Neem oil NE | — | Larvicidal (immersion) | — | 11.75 mg/L |

***Expressed as percentage LC₅₀; converted to ppm (10,030 ppm).**

- **Contact:** Direct application or exposure to treated surfaces/medium; **Fumigant:** Volatile exposure in a sealed/vapor phase system; **Larvicidal (immersion):** Immersion or introduction of larvae to treated aqueous medium.

**References**

**Abd El Salam, K. M., Abou El-Soud, G. M., Abd El Salam, M. M., Abd El-Naby, S. S., Abdel-Rheim, K. H., Lamlom, S. F., & Abdel-Megeed, A. (2024).** Natural nano-emulsions: A sustainable solution for rice weevil control in stored paddy rice. *Journal of Stored Products Research*, 109, 102451. https://doi.org/10.1016/j.jspr.2024.102451

**Abd El-Naby, S. I., Mahmoud, F. H., El-Habal, N. A., Abdou, M. S., & Abdel-Rheim, K. H. (2023).** Efficacy of prepared castor oil nanoemulsion formulation against rice weevil *Sitophilus oryzae* on stored wheat grain and its acute. *Egyptian Scientific Journal of Pesticides*, 6, 33-34.

**Abd El‑Naby, S. S., Mahmoud, F. H., Abdou, M. S., El‑Habal, N. A., Jadalla, N. M., & Abdel‑Halim, K. Y. (2024).** Nano‑emulsion form of castor seed oil as a new alternative to control *Tribolium castaneum* and *Oryzaephilus surinamensis* and its histological alteration in rats. *Archives of Phytopathology and Plant Protection, 56*, 1391–1410. https://doi.org/10.1080/03235408.2023.2289212

**Abdul Qayyum, K., Tahir, H. M., Ali, A., Tariq, M., Mushtaq, B., Muzamil, A., … Ghaffar, A. (2025).** Insecticidal activity of polyethylene glycol nanocapsules of clove essential oil against *Sitophilus oryzae* and *Tribolium castaneum*. *Journal of Zoology and Systematics, 3*(1), 47–59. https://doi.org/10.56946/jzs.v3i1.562

**Anjali, C. H., Sharma, Y., Mukherjee, A., & Chandrasekaran, N. (2012).** Neem oil (Azadirachta indica) nanoemulsion—a potent larvicidal agent against *Culex quinquefasciatus*. *Pest Management Science*, 68(2), 158–163. https://doi.org/10.1002/ps.2233

**Avinash M, YC Vishwanath, NK Hegde, Ramanagouda Hadlageri, Mahantesh Naika BN, Sadananda GK and Sudheesh Kulkarni (2025).** GC-MS analysis of cinnamon (Cinnamomum verum J. Presl) bark essential oil under hill zone of Karnataka. International Journal of Research in Agronomy, Vol. 8, Issue 4, Part B (2025). Doi: <https://doi.org/10.33545/2618060X.2025.v8.i4b.2744>

**Cantó Catalá, A., & Blázquez Ferrer, M. A. (2017).** Standardization of commercial cinnamon essential oils by gas chromatography–mass spectrometry analysis. *Nereis. Revista Iberoamericana Interdisciplinar de Métodos, Modelización y Simulación*, (9), 86–90.

**Chaieb, K., Hajlaoui, H., Zmantar, T., Kahla-Nakbi, A. B., Rouabhia, M., Mahdouani, K., & Bakhrouf, A. (2007).** The chemical composition and biological activity of clove essential oil, Eugenia caryophyllata (Syzigium aromaticum L. Myrtaceae): A short review. *Phytotherapy Research, 21*(6), 501–506. <https://doi.org/10.1002/ptr.2124>

**ESCOP. (2003).** *ESCOP monographs: The scientific foundation for herbal medicinal products* (2nd ed.). Thieme.

**Fadel, H.H.M, El-Ghorab, A.H., Hussein, A.M.S., El-Massry, K.F., Lotfy, Sh.N., Soliman, T.N. (2020).** Correlation between chemical composition and radical scavenging activity of 10 commercial essential oils: Impact of microencapsulation on functional properties of essential oils. Arabian Journal of Chemistry (2020), <https://doi.org/10.1016/j.arabjc.2020.06.034>

**Haro-González JN, Castillo-Herrera GA, Martínez-Velázquez M, Espinosa-Andrews H.** (**2021**) Clove essential oil (Syzygium aromaticum L. Myrtaceae): extraction, chemical composition, food applications, and essential bioactivity for human health. Molecules.;26(21):6387. doi: <https://doi.org/10.3390/molecules26216387>

**Heydari, M., Amirjani, A., Bagheri, M., Sharifian, I., & Sabahi, Q. (2020).** Eco‑friendly pesticide based on peppermint oil nanoemulsion: Preparation, physicochemical properties, and its aphicidal activity against cotton aphid. *Environmental Science and Pollution Research International, 27*(6), 6667–6679. https://doi.org/10.1007/s11356-019-07332-y

**Industrial Crops and Products. (2013).** Analysis and evaluation of essential oil components of cinnamon barks using GC–MS and FTIR spectroscopy. *Industrial Crops and Products, 41*, 269–278. <https://doi.org/10.1016/j.indcrop.2012.04.056>

**Iqbal, L. Z., Ikhtiar, F., Farooq, M. U., Faraz, M. F., Riaz, T., Haider, A., & Ullah, R. H. (2025).** Formulation and evaluation of *Syzygium aromaticum* essential oil nanoemulsion: Effects on *Tribolium castaneum*, wheat growth, and molecular docking for pest control. *Brazilian Journal of Science*, 4(3), 1–24. https://doi.org/10.14295/bjs.v4i3.712

**Jasman, A. K., Slomy, A. K., & Khaleel, A. I. (2025).** Nanoemulsion-enhanced insecticidal activity of *Cymbopogon citratus* and *Mentha longifolia* essential oils against the red flour beetle (*Tribolium castaneum*). *Journal of Stored Products Research*, 114, 102759. https://doi.org/10.1016/j.jspr.2025.102759

**Jirovetz, L., Buchbauer, G., Stoilova, I., Stoyanova, A., Krastanov, A., & Schmidt, E. (2006).** Chemical composition and antioxidant properties of clove leaf essential oil. *Journal of Agricultural and Food Chemistry, 54*(17), 6303–6307. <https://doi.org/10.1021/jf060608c>

**Kavallieratos, N. G., Bonacucina, G., Nika, E. P., Skourti, A., Georgakopoulou, S. K. C., Filintas, C. S., & Benelli, G. (2023).** The type of grain counts: Effectiveness of three essential oil-based nanoemulsions against *Sitophilus oryzae*. *Plants*, 12, 813. https://doi.org/10.3390/plants12040813

**Lokesh, M., Panneerselvam, A., Sreekrishnakumar, A. K., Anand, A., Gowda, T. S., & Vendan, S. E. (2024).** Intensification of biofumigant stability: preparation of peppermint-piperitone nanoemulsion for effective fumigation against stored-product beetles. *Journal of Food Science and Technology*, 61, 1300–1309. https://doi.org/10.1007/s13197-024-06181-z

**Mutlu M, Bingol Z, Uc EM, Köksal E, Goren AC, Alwasel SH, Gulcin İ.** Comprehensive Metabolite Profiling of Cinnamon (*Cinnamomum zeylanicum*) Leaf Oil Using LC-HR/MS, GC/MS, and GC-FID: Determination of Antiglaucoma, Antioxidant, Anticholinergic, and Antidiabetic Profiles. Life (Basel). 2023 Jan 3;13(1):136. doi: <https://doi.org/10.3390/life13010136>

**Narasimman, M., Natesan, V., Mayakrishnan, V., Rajendran, J., Venkatesan, A., & Kim, S.-J. (2022).** Preparation and optimization of peppermint (*Mentha piperita*) essential oil nanoemulsion with effective herbal larvicidal, pupicidal, and ovicidal activity against *Anopheles stephensi*. *Current Pharmaceutical Biotechnology*, 23(11), 1367–1376. https://doi.org/10.2174/1389201023666211215125621

**Rohimatun, R., Aisyah, M. D. N., Rismayani, R., Rizal, M., Noveriza, R., & Mardiningsih, T. L. (2024).** Evaluation of citronella oil nanoemulsion formulation against the insect-stored pest *Callosobruchus maculatus* (Fab.) (Coleoptera: Bruchidae). *Journal of Plant Protection Research*, 64(3), 288–297. https://doi.org/10.24425/jppr.2024.151256

**Singh, G., Maurya, S., de Lampasona, M. P., & Catalan, C. (2007).** A comparison of chemical, antioxidant and antimicrobial studies of cinnamon leaf and bark volatile oils, oleoresins and their constituents. *Food and Chemical Toxicology, 45*(9), 1650–1661. <https://doi.org/10.1016/j.fct.2007.02.031>

**Soković, M., Glamočlija, J., Marin, P. D., Brkić, D., & van Griensven, L. J. L. D. (2009).** Antibacterial effects of the essential oils of commonly consumed medicinal herbs using an in vitro model. *Molecules, 14*(1), 238–249. <https://doi.org/10.3390/molecules14010238>

**World Health Organization. (1999).** *WHO monographs on selected medicinal plants* (Vol.1). WHO.
